# Global influenza surveillance systems to detect the spread of influenza-negative influenza-like illness during the COVID-19 pandemic: Time series outlier analyses from 2015–2020

**DOI:** 10.1371/journal.pmed.1004035

**Published:** 2022-07-19

**Authors:** Natalie L. Cobb, Sigrid Collier, Engi F. Attia, Orvalho Augusto, T. Eoin West, Bradley H. Wagenaar

**Affiliations:** 1 Division of Pulmonary, Critical Care and Sleep Medicine, Department of Medicine, University of Washington, Seattle, Washington, United States of America; 2 Division of Dermatology, Department of Medicine, University of Washington, Seattle, Washington, United States of America; 3 Veterans Affairs Puget Sound Health Care System, Seattle, Washington, United States of America; 4 Department of Global Health, University of Washington, Seattle, Washington, United States of America; 5 Department of Microbiology and Immunology, Faculty of Tropical Medicine, Mahidol University, Bangkok, Thailand; 6 Department of Epidemiology, University of Washington, Seattle, Washington, United States of America; Universitair Medisch Centrum Utrecht, NETHERLANDS

## Abstract

**Background:**

Surveillance systems are important in detecting changes in disease patterns and can act as early warning systems for emerging disease outbreaks. We hypothesized that analysis of data from existing global influenza surveillance networks early in the COVID-19 pandemic could identify outliers in influenza-negative influenza-like illness (ILI). We used data-driven methods to detect outliers in ILI that preceded the first reported peaks of COVID-19.

**Methods and findings:**

We used data from the World Health Organization’s Global Influenza Surveillance and Response System to evaluate time series outliers in influenza-negative ILI. Using automated autoregressive integrated moving average (ARIMA) time series outlier detection models and baseline influenza-negative ILI training data from 2015–2019, we analyzed 8,792 country-weeks across 28 countries to identify the first week in 2020 with a positive outlier in influenza-negative ILI. We present the difference in weeks between identified outliers and the first reported COVID-19 peaks in these 28 countries with high levels of data completeness for influenza surveillance data and the highest number of reported COVID-19 cases globally in 2020. To account for missing data, we also performed a sensitivity analysis using linear interpolation for missing observations of influenza-negative ILI. In 16 of the 28 countries (57%) included in this study, we identified positive outliers in cases of influenza-negative ILI that predated the first reported COVID-19 peak in each country; the average lag between the first positive ILI outlier and the reported COVID-19 peak was 13.3 weeks (standard deviation 6.8). In our primary analysis, the earliest outliers occurred during the week of January 13, 2020, in Peru, the Philippines, Poland, and Spain. Using linear interpolation for missing data, the earliest outliers were detected during the weeks beginning December 30, 2019, and January 20, 2020, in Poland and Peru, respectively. This contrasts with the reported COVID-19 peaks, which occurred on April 6 in Poland and June 1 in Peru. In many low- and middle-income countries in particular, the lag between detected outliers and COVID-19 peaks exceeded 12 weeks. These outliers may represent undetected spread of SARS-CoV-2, although a limitation of this study is that we could not evaluate SARS-CoV-2 positivity.

**Conclusions:**

Using an automated system of influenza-negative ILI outlier monitoring may have informed countries of the spread of COVID-19 more than 13 weeks before the first reported COVID-19 peaks. This proof-of-concept paper suggests that a system of influenza-negative ILI outlier monitoring could have informed national and global responses to SARS-CoV-2 during the rapid spread of this novel pathogen in early 2020.

## Abstract 2

Natalie L Cobb and colleagues use routine influenza surveillance data to detect outliers in influenza-like-illness during the COVID-19 pandemic.

## Author summary

**Why was this study done?:**

- Early detection of respiratory viral outbreaks, such as SARS-CoV-2, is key for public health response and mitigation measures.
- In this study, we used routine influenza surveillance data to detect outliers in influenza-like illness (ILI) during the COVID-19 pandemic that could suggest spread of SARS-CoV-2.
- We hypothesized that using data-driven methods would identify increased case counts of influenza-negative ILI prior to reported peaks of COVID-19.

**What did the researchers do and find?:**

- We used routine influenza surveillance data from the World Health Organization’s FluNet and applied automated outlier detection methods to identify outliers in influenza-negative ILI in 2020 across 28 countries.
- In 16 countries, we detected outliers that preceded the first reported COVID-19 peaks, with an average lag time of 13.3 weeks.
- In 7 countries, the week of the first outlier changed when accounting for missing data in the models.

**What do these findings mean?:**

- This study serves as a proof of concept and suggests a potential role for the use of automated data monitoring and outlier detection systems to identify outbreaks in respiratory viral illness.
- These findings also highlight the importance of strengthening routine disease surveillance networks to enhance our ability to identify novel diseases and inform public health responses on a global scale.

## Introduction

Severe acute respiratory syndrome coronavirus 2 (SARS-CoV-2) was first reported from a cluster of pneumonia patients in Wuhan, China, during late December 2019 [1,2]. Over the following months, the virus rapidly spread across the globe, and on March 11, 2020, a pandemic was declared by the World Health Organization (WHO) [3]. During the early weeks of the pandemic, diagnostic testing for SARS-CoV-2 was limited, and low rates of testing likely resulted in under-detection of cases and contributed to global spread [4,5], especially in low- and middle-income countries (LMICs), which were less likely to have access to diagnostic tests. Since the outbreak first began, studies in high-income countries (HICs) have demonstrated that community transmission was occurring in the weeks preceding the first known peaks of coronavirus disease 2019 (COVID-19) [5–8]. Moreover, data from influenza surveillance networks in France and the United States have suggested that in early 2020 there was a substantial increase in cases of influenza-like illness (ILI) that coincided with a rise in COVID-19 cases and may have represented undetected SARS-CoV-2 infection [9,10]. Studies in New York City and the Lombardy region of Italy, both of which experienced major outbreaks early in the pandemic, demonstrated a high rate of SARS-CoV-2 positivity among cases of ILI during March 2020 [11,12]. In New York City, using laboratory-based sentinel surveillance, there were over 1,000 estimated undetected SARS-CoV-2 cases in early March [12]. Limited information exists on whether similar patterns may have occurred in LMICs that could provide insight into the extent of global spread of COVID-19 in early 2020.

Leveraging existing and long-running influenza surveillance systems may provide insight into the early spread of SARS-CoV-2 globally and serve to enhance monitoring for emerging non-influenza respiratory viral pathogens. The WHO Global Influenza Surveillance and Response System (GISRS) is a network comprising national influenza centers and WHO collaborating centers and laboratories across 123 WHO member states that collect respiratory specimens for laboratory influenza testing [13]. Samples are collected from individuals presenting to participating centers with symptoms of ILI or severe acute respiratory infection [14]. Data from the national influenza centers, influenza reference laboratories, and WHO regional databases are made available through FluNet, a web-based tool used for monitoring global trends in influenza [15–18].

In this study, we evaluate outliers in influenza-negative ILI in 2020 compared to trends over the previous 5 years among countries with established ILI surveillance systems and a high incidence of COVID-19. We hypothesized that automated outlier detection methods would detect outliers in influenza-negative ILI during 2020 that preceded the first peaks of SARS-CoV-2. This study is intended as a proof of concept for the use of continuous automated global detection of influenza-negative ILI outliers as a potential early warning system for novel circulating viral pathogens, which could be especially vital for LMICs, which often have limited access to the rapid development and deployment of diagnostic tests for novel viral pathogens such as SARS-CoV-2.

## Methods

### Data sources

All data were obtained from publicly available sources. Weekly and cumulative COVID-19 cases by country were obtained from the WHO COVID-19 dashboard [19]. Cases of COVID-19 are based on WHO case definitions and laboratory-confirmed cases, which are reported to the WHO and include monitoring by ministries of health [20]. Data for influenza-negative ILI cases between January 2015 and December 2020 were obtained from FluNet, a web-based tool that reports influenza surveillance data from national influenza centers, influenza reference laboratories, and WHO regional databases of the GISRS [15]. FluNet data are updated weekly and include the number of respiratory specimens processed, laboratory-confirmed influenza samples, and influenza-negative samples among individuals seeking care for ILI or severe acute respiratory infection [14,15]. The WHO definition of ILI is a measured fever ≥ 38 degrees Celsius and cough, with onset of these symptoms within the last 10 days [21]. Severe acute respiratory infection is defined by symptoms of ILI and hospitalization [21]. Datasets from FluNet and the WHO COVID-19 dashboard were linked by country and week according to the International Organization for Standardization (ISO) system. There were 314 weeks observed per country for 2015–2020. In 2020, there were 53 ISO weeks, which began the week of December 30, 2019 (week 1), and ended on January 3, 2021 (week 53).

### Selection criteria

To ensure a representative sample of countries across income groups, we selected the top 40 countries with highest cumulative number of COVID-19 cases through January 3, 2021 (ISO week 53), stratified by World Bank income classification (low income, lower middle income, upper middle income, and high income), which are listed in S1 Text. Countries were excluded if they had greater than 25% missing data based on the calculation of an overall completeness score. The score, which was adapted from a previous study [22], was calculated as the average percentage of data available for specimens processed, influenza-positive specimens, and weekly data for 2020 (S2 Text). Using this method, the following 28 countries were eligible for inclusion in the analysis: Afghanistan, Argentina, Bangladesh, Bolivia, Brazil, Colombia, Democratic Republic of the Congo, France, Germany, India, Indonesia, Iran, Madagascar, Mexico, Mozambique, Nepal, Netherlands, Peru, the Philippines, Poland, Republic of Moldova, Russia, South Africa, Spain, Uganda, Ukraine, United Kingdom, and United States.

### Definitions

To identify the first COVID-19 peak in each country, we evaluated graphs of weekly COVID-19 cases from the WHO data. The peak was defined as the first week with the highest number of cases within a period of rising cases followed by a decrease or plateau over a duration of at least 3 weeks. The minimum threshold for a peak was at least 100 cases per week.

Data for weekly cases of influenza-negative ILI were abstracted as reported in FluNet. Cases are reported in absolute values. If influenza-negative ILI was not reported, a value was calculated according to the weekly number of specimens processed minus the weekly number of influenza-positive specimens. Values less than 0 were considered erroneous and treated as missing data.

### Statistical analysis

The aim of this study was to identify positive outliers in influenza-negative ILI during the first year of the COVID-19 pandemic using automated data-driven time series methods. Our a priori analysis plan included (1) stratifying countries by income group and identifying the countries with the highest number of COVID-19 cases in 2020, (2) exploring missing data and excluding countries with high levels of missingness, (3) utilizing automated outlier detection methods to identify positive outliers in influenza-negative ILI during 2020, (4) evaluating the time difference between detected ILI outliers and reported COVID-19 peaks, and (5) performing a sensitivity analysis using interpolation for missing data. To address comments during peer review, we subsequently performed model fit diagnostics to evaluate the autocorrelation of residuals for each fitted model.

Descriptive statistics, including the median weekly influenza-negative ILI case counts, are reported by World Bank income classification. To identify outliers in time series trends, we used a data-driven approach based on autoregressive integrated moving average (ARIMA) modeling with the “tsoutliers” package in R [23]. The “tso” function uses automated ARIMA models and selects the best-fitting model using the conditional sum of squares and maximum likelihood [23,24]. Differencing terms, to address data stationarity, are determined by a unit root test [24]. Outliers are iteratively detected based on a critical value according to sample size [23]. In this analysis, we evaluated additive outliers, which are defined by an acute change at a single time point without a change in subsequent observations, and temporary change outliers, which are defined as a transient change that affects more than 1 consecutive observation before returning to baseline. Seasonal level shifts were included as well, which evaluate a sustained change in observations accounting for seasonal patterns. We excluded missing observations from the primary analysis (Model 1). The primary outcome of interest was the first week with a positive outlier in 2020, in which the observed number of influenza-negative ILI cases exceeded the expected trend for each country. We also report all positive outliers detected during the time series in S3 Text. We calculated the difference in weeks between the first positive outlier and the first COVID-19 peak for countries where the detected outlier preceded the reported peak. To account for the impact of missing data, we also performed a sensitivity analysis in which we repeated the analysis as described but using linear interpolation for missing values of influenza-negative ILI (Model 2). Lastly, for each fitted model (Model 1), we graphed the autocorrelation function and partial autocorrelation function of residuals. We performed the Ljung–Box test to evaluate residual autocorrelation. All analyses were conducted in R version 4.1.2.

## Results

Among the 28 countries included in this study, 4.7% (413/8,792) of observations for influenza-negative ILI were missing. The number of missing observations by country is provided in S1 Table. The median number of reported influenza-negative ILI cases per week across all countries from 2015 to 2020 was 69, with an interquartile range (IQR) of 20–268. In high-income countries (HICs), the median was 206.5 (IQR 33–1,553.5); in upper-middle-income countries, 164 (IQR 68–528); in lower-middle-income countries, 33 (IQR 15–99.3); and in low-income countries, 19 (IQR 9–38). In HICs, the median number of influenza-negative ILI cases per week in 2020 was 274, compared to 194 in 2015–2019 (Table 1).

**Table 1:** Counts of influenza-negative ILI by income group and data missingness.

Measure	Overall	HICs	U-MICs	L-MICs	LICs
**Weekly influenza-negative ILI cases, median (IQR)**					
2015–2020	69 (20–268)	206.5 (33–1,553.5)	164 (68–528)	33 (15–99.3)	19 (9–38)
2015–2019	71 (23–257.8)	194 (33–1,452)	165 (72–491.2)	34 (17–103)	20 (10–39)
2020	54 (8–381)	274 (16.5–1,854)	155 (30–857)	23 (3–83.5)	15 (4–30)
**Missing observations for influenza-negative ILI, *n/N* (%)**	413/8,792 (4.7)	178/2,198 (8.1)	85/2,826 (3.0)	70/2,198 (3.2)	80/1,570 (5.1)

HICs, high-income countries; ILI, influenza-like illness; IQR, interquartile range; L-MICs, lower-middle-income countries; LICs, low-income countries; U-MICs, upper-middle-income countries.

### Outliers in influenza-negative ILI during 2020

**Fig 1:**
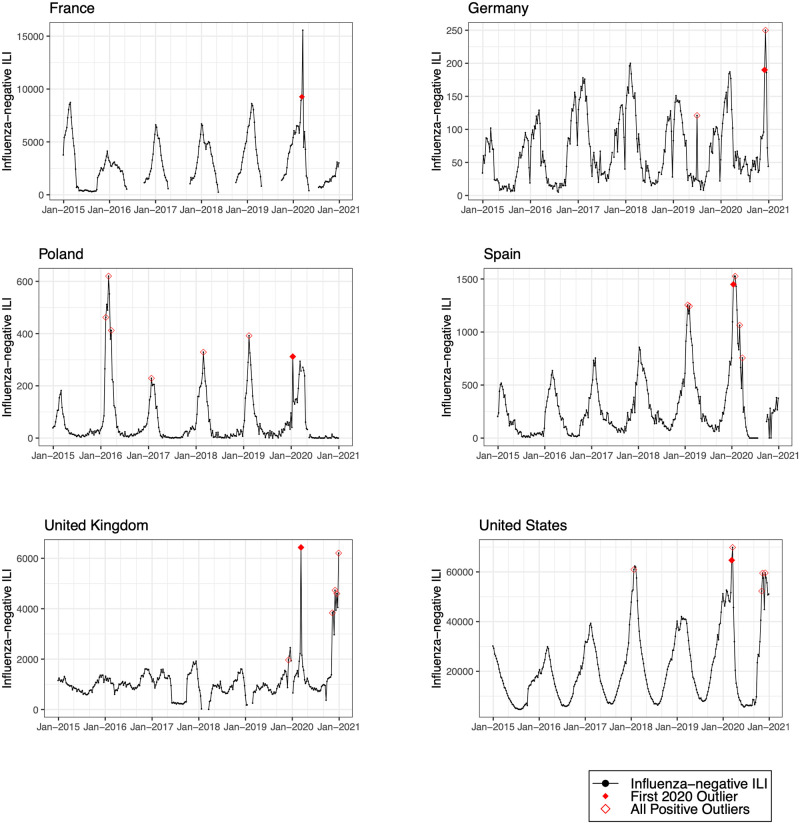
Observed trends in influenza-negative influenza-like illness (ILI) and detected outliers in high-income countries. Reported cases of influenza-negative ILI in each country between 2015 and 2020. Open red diamonds indicate all detected positive outliers, and filled red diamonds indicate the first outlier in 2020.

**Fig 2:**
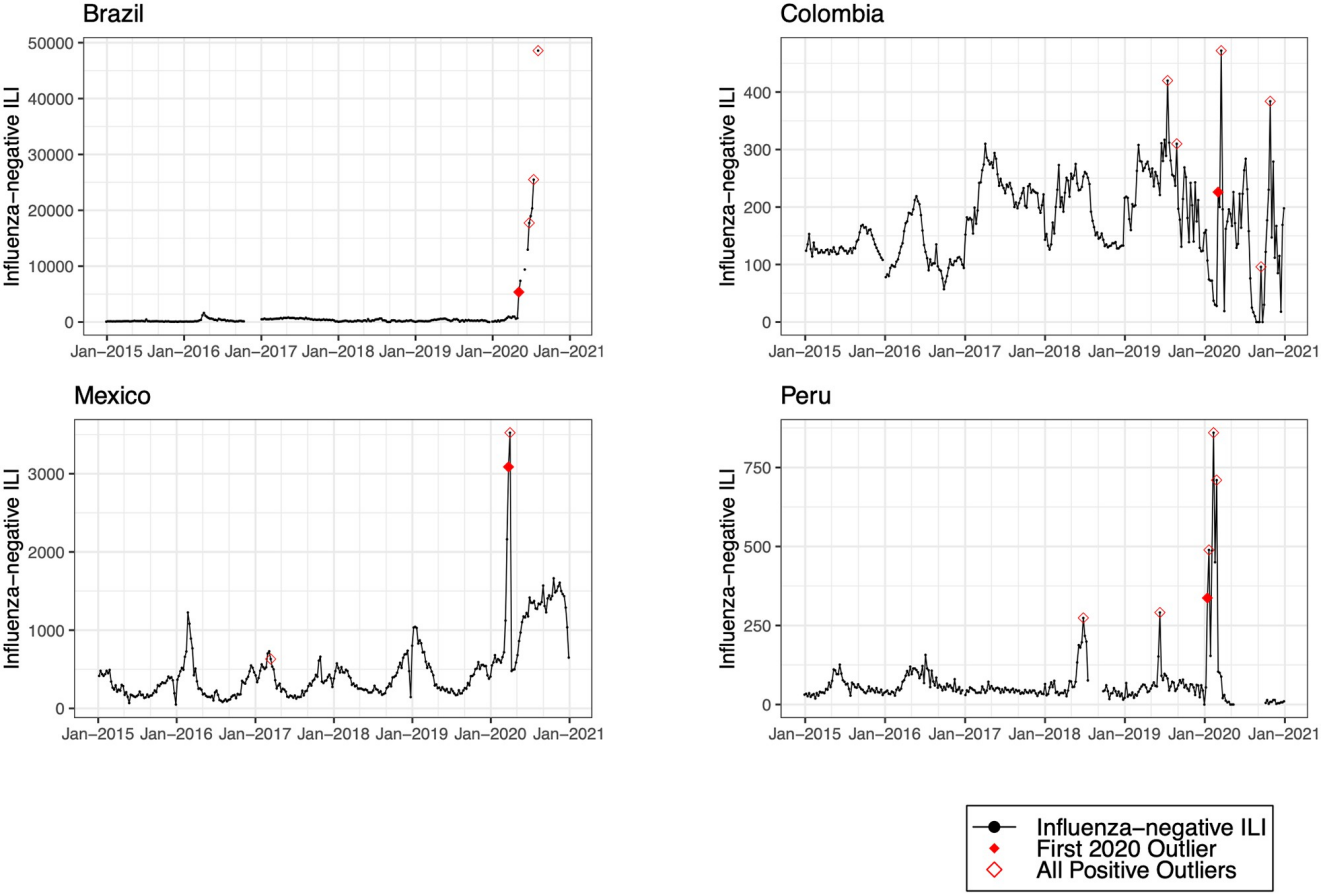
Observed trends in influenza-negative influenza-like illness (ILI) and detected outliers in upper-middle-income countries. Reported cases of influenza-negative ILI in each country between 2015 and 2020. Open red diamonds indicate all detected positive outliers, and filled red diamonds indicate the first outlier in 2020.

**Fig 3:**
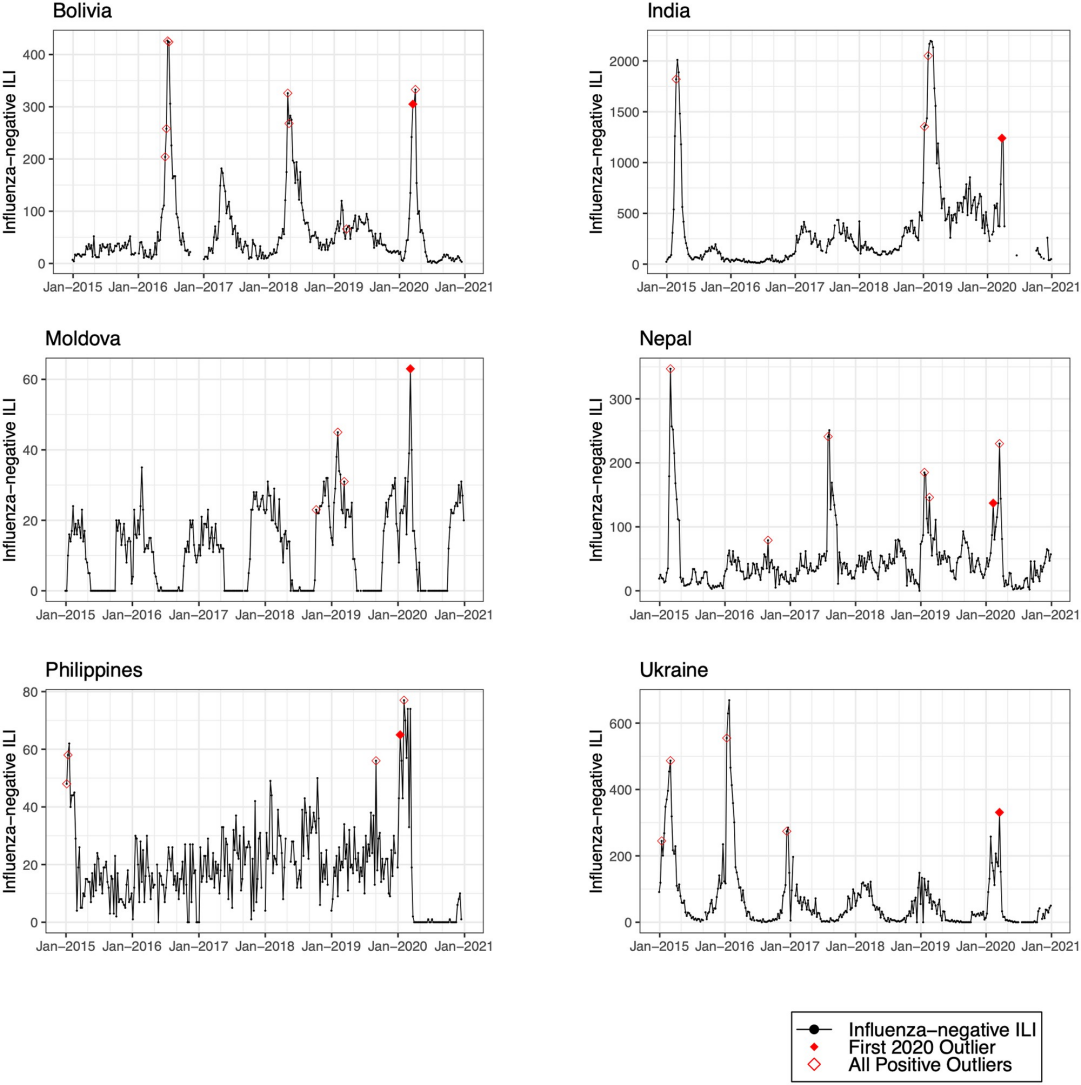
Observed trends in influenza-negative influenza-like illness (ILI) and detected outliers in lower-middle-income countries. Reported cases of influenza-negative ILI in each country between 2015 and 2020. Open red diamonds indicate all detected positive outliers, and filled red diamonds indicate the first outlier in 2020.

**Fig 4:**
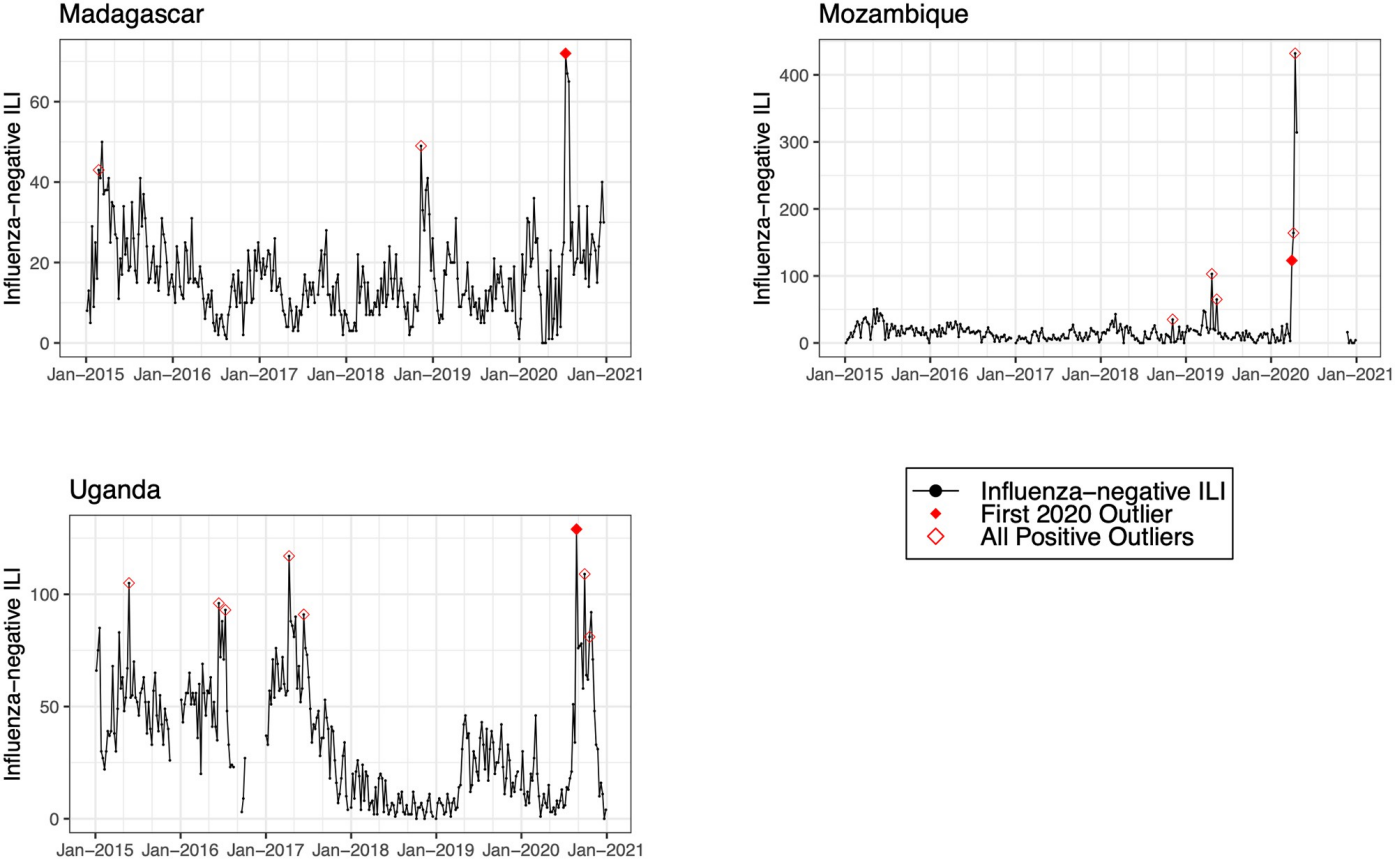
Observed trends in influenza-negative influenza-like illness (ILI) and detected outliers in low-income countries. Reported cases of influenza-negative ILI in each country between 2015 and 2020. Open red diamonds indicate all detected positive outliers, and filled red diamonds indicate the first outlier in 2020.

**Fig 5:**
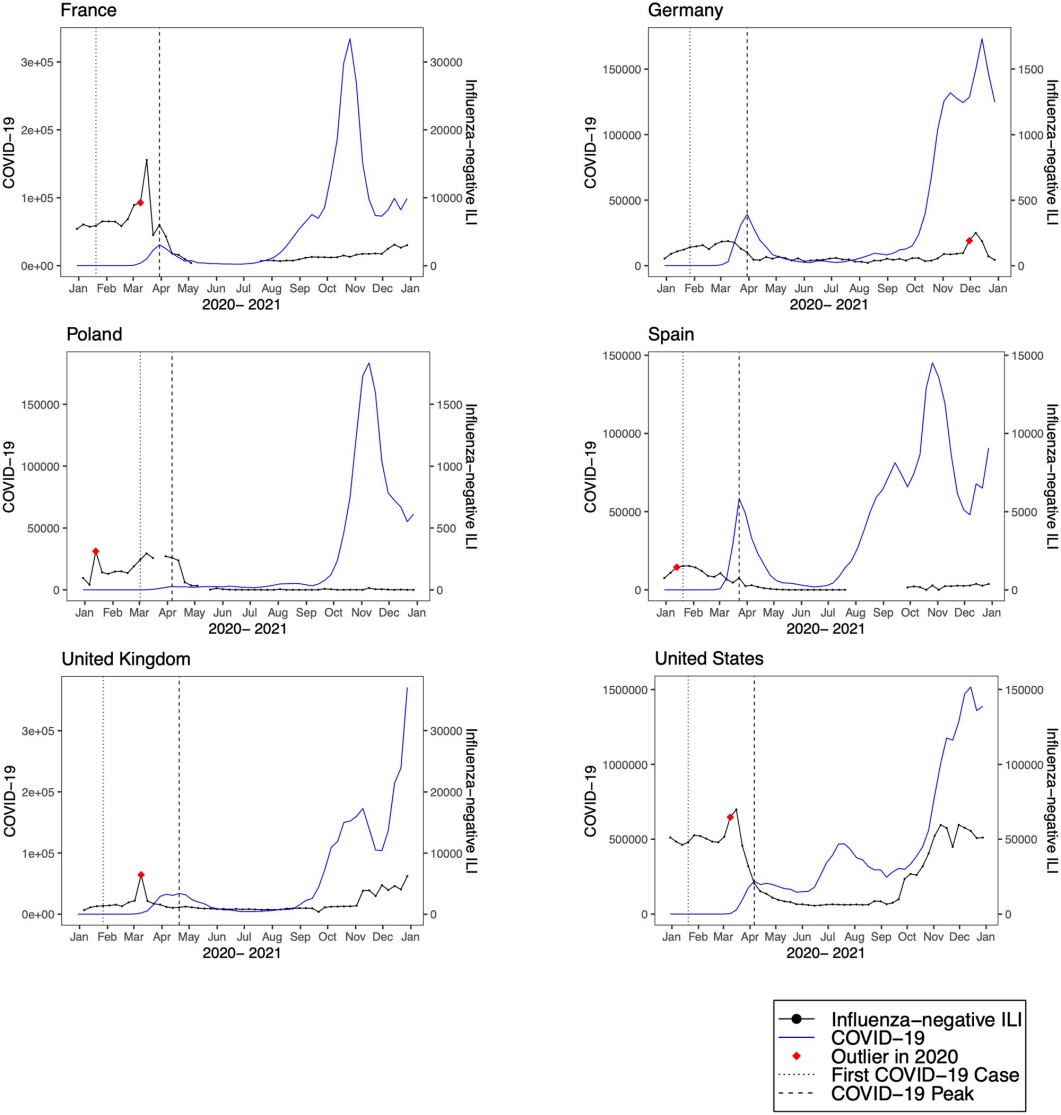
Observed trends in COVID-19 compared to influenza-negative influenza-like illness (ILI) in high-income countries. Trends in reported COVID-19 cases (blue line) and influenza-negative ILI cases (black line) during 2020. The vertical dotted black line indicates the week of the first COVID-19 case, the vertical dashed line indicates the week of the first COVID-19 peak, and the red diamond indicates the first detected influenza-negative ILI outlier in 2020.

**Fig 6:**
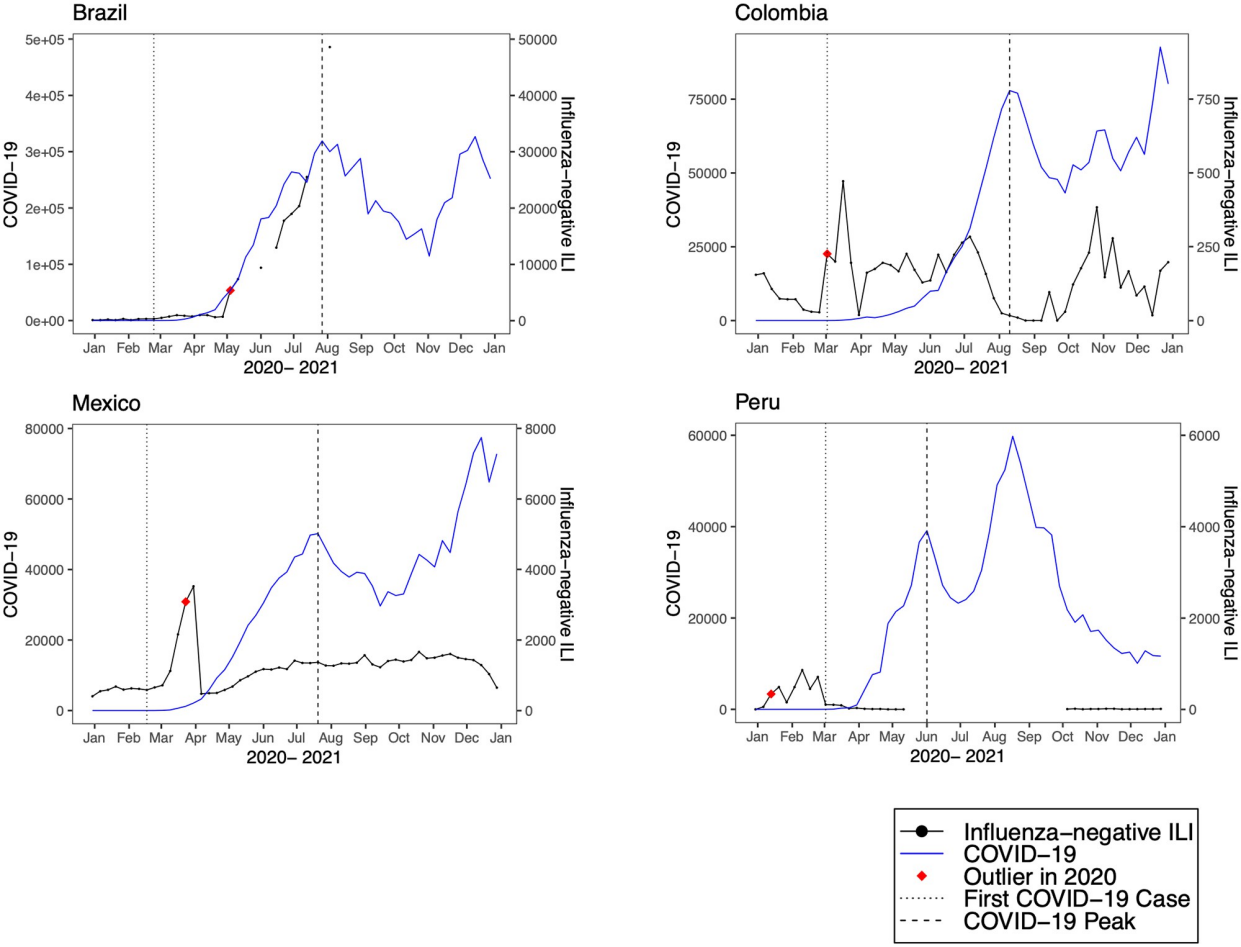
Observed trends in COVID-19 compared to influenza-negative influenza-like illness (ILI) in upper-middle-income countries. Trends in reported COVID-19 cases (blue line) and influenza-negative ILI cases (black line) during 2020. The vertical dotted black line indicates the week of the first COVID-19 case, the vertical dashed line indicates the week of the first COVID-19 peak, and the red diamond indicates the first detected influenza-negative ILI outlier in 2020.

**Fig 7:**
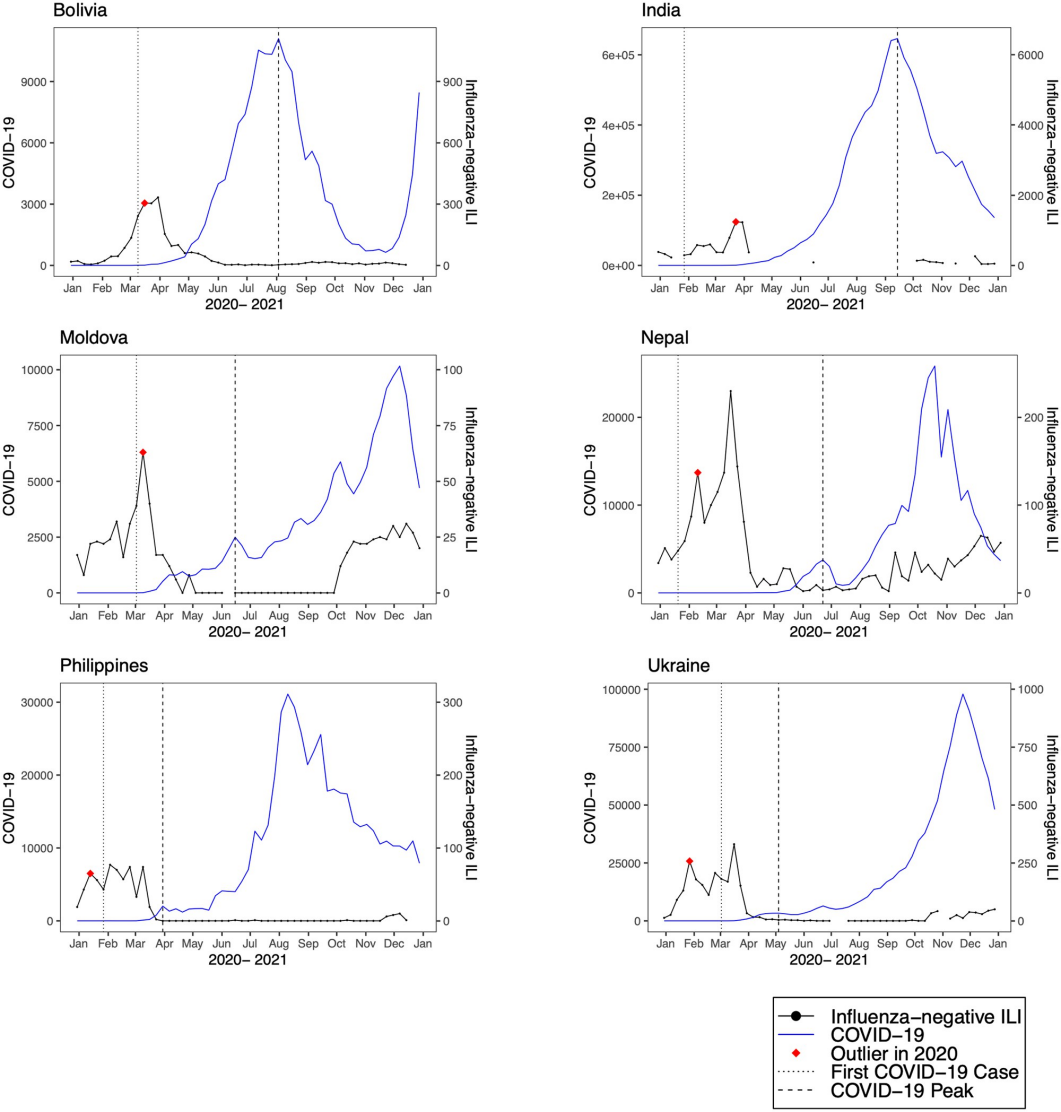
Observed trends in COVID-19 compared to influenza-negative influenza-like illness (ILI) in lower-middle-income countries. Trends in reported COVID-19 cases (blue line) and influenza-negative ILI cases (black line) during 2020. The vertical dotted black line indicates the week of the first COVID-19 case, the vertical dashed line indicates the week of the first COVID-19 peak, and the red diamond indicates the first detected influenza-negative ILI outlier in 2020.

**Fig 8:**
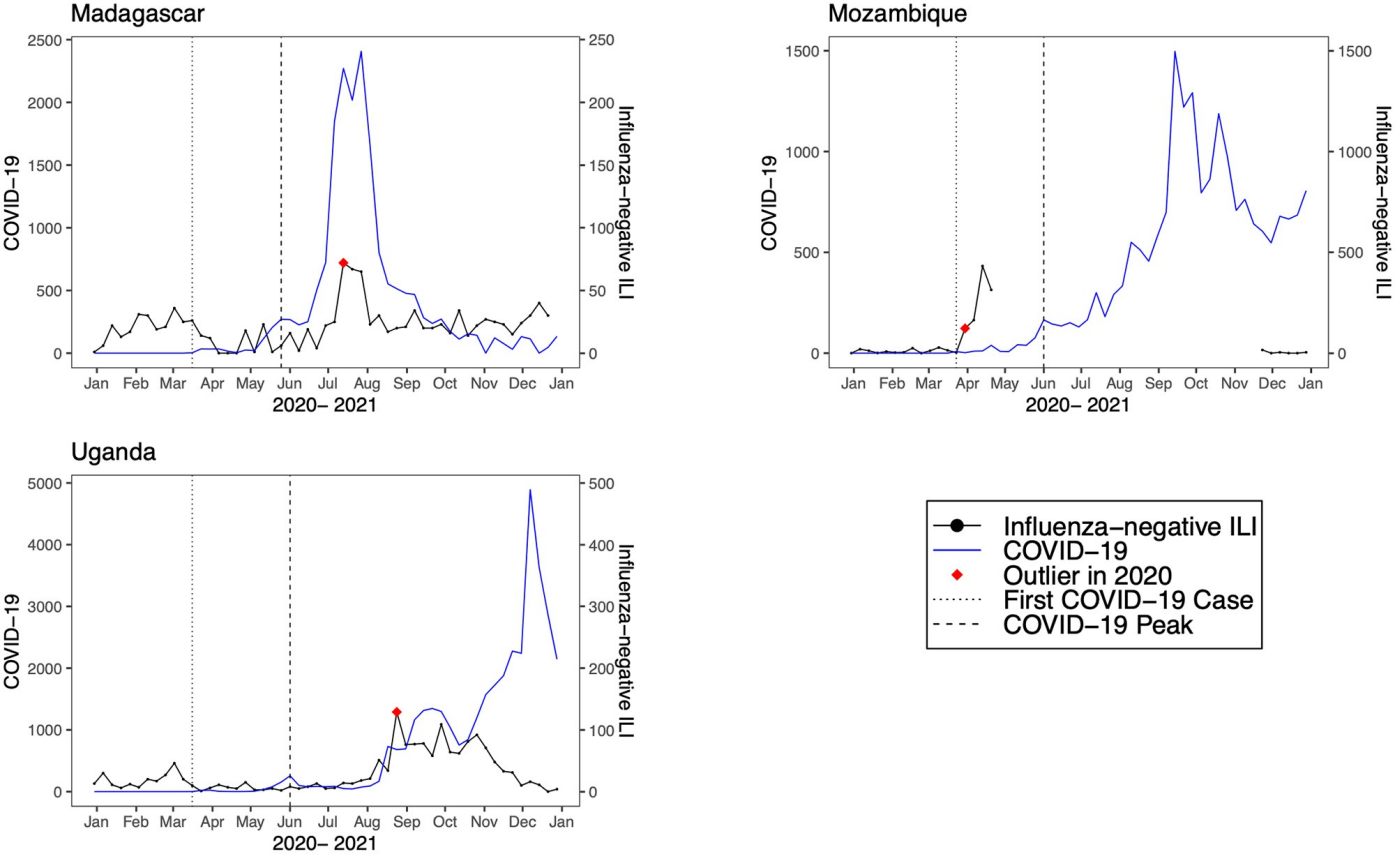
Observed trends in COVID-19 compared to influenza-negative influenza-like illness (ILI) in low-income countries. Trends in reported COVID-19 cases (blue line) and influenza-negative ILI cases (black line) during 2020. The vertical dotted black line indicates the week of the first COVID-19 case, the vertical dashed line indicates the week of the first COVID-19 peak, and the red diamond indicates the first detected influenza-negative ILI outlier in 2020.

We detected positive outliers during 2020 in 19 countries (68%). In 6 countries (21%; Afghanistan, Argentina, Bangladesh, Indonesia, Netherlands, and South Africa), no positive outliers were detected during 2020, and in 3 countries (11%; Democratic Republic of the Congo, Iran, and Russia), outlier analysis was limited by model non-convergence. For the 19 countries in which a positive outlier was detected, the outlier predated the first reported COVID-19 peak in 16 countries (84%) (Table 2). In 3 countries (Germany, Madagascar, and Uganda), the outlier occurred after the first reported COVID-19 peak. Figs 1–8 present the observed trends in influenza-negative ILI and COVID-19 for countries with positive outliers. Observed trends for countries without detected outliers in 2020 are presented in S1 and S2 Figs. The identified outliers in each country were either additive outliers (7/19, 37%) or temporary change outliers (12/19, 63%).

**Table 2:** Comparison by week of the first detected influenza-negative ILI time series outlier and the first reported COVID-19 case and peak by country.

Country	Week of detected time series outlier (type)*	Week of first reported COVID-19 case	Week of first reported COVID-19 peak	Difference in weeks between outlier and reported peak
**HICs**				
France	09-Mar-2020 (TC)	20-Jan-2020	30-Mar-2020	3
Germany	30-Nov-2020 (TC)	27-Jan-2020	30-Mar-2020	−35
Netherlands	—	24-Feb-2020	06-Apr-2020	—
Poland	13-Jan-2020 (AO)	02-Mar-2020	06-Apr-2020	12
Spain	13-Jan-2020 (TC)	20-Jan-2020	23-Mar-2020	10
United Kingdom	09-Mar-2020 (AO)	27-Jan-2020	20-Apr-2020	6
United States	09-Mar-2020 (TC)	20-Jan-2020	06-Apr-2020	4
**U-MICs**				
Argentina	—	02-Mar-2020	19-Oct-2020	—
Brazil	04-May-2020 (TC)	24-Feb-2020	27-Jul-2020	12
Colombia	02-Mar-2020 (TC)	02-Mar-2020	10-Aug-2020	23
Indonesia	—	02-Mar-2020	21-Sep-2020	—
Mexico	23-Mar-2020 (TC)	17-Feb-2020	20-Jul-2020	17
Peru	13-Jan-2020 (AO)	02-Mar-2020	01-Jun-2020	20
South Africa	—	02-Mar-2020	13-July-2020	—
**L-MICs**				
Bangladesh	—	02-Mar-2020	22-Jun-2020	—
Bolivia	16-Mar-2020 (TC)	09-Mar-2020	03-Aug-2020	20
India	23-Mar-2020 (TC)	27-Jan-2020	14-Sep-2020	25
Republic of Moldova	09-Mar-2020 (AO)	02-Mar-2020	15-Jun-2020	14
Nepal	10-Feb-2020 (AO)	20-Jan-2020	22-Jun-2020	19
Philippines	13-Jan-2020 (TC)	27-Jan-2020	30-Mar-2020	11
Ukraine	16-Mar-2020 (AO)	02-Mar-2020	04-May-2020	7
**LICs**				
Afghanistan	—	24-Feb-2020	01-Jun-2020	—
Madagascar	13-Jul-2020 (TC)	16-Mar-2020	25-May-2020	−7
Mozambique	30-Mar-2020 (AO)	23-Mar-2020	01-Jun-2020	9
Uganda	24-Aug-2020 (TC)	16-Mar-2020	01-Jun-2020	−12

*Additive outlier (AO) or temporary change outlier (TC).

HICs, high-income countries; L-MICs, lower-middle-income countries; LICs, low-income countries; U-MICs, upper-middle-income countries.

The earliest outliers occurred during the week of January 13, 2020, in Peru, the Philippines, Poland, and Spain. Among the countries with positive outliers that predated the first reported COVID-19 peak, time series outliers were detected on average 13.3 weeks prior to the reported COVID-19 peak (standard deviation 6.8). Seven countries demonstrated a lag between the first detected outlier and reported COVID-19 peak of greater than 12 weeks (Bolivia, Colombia, India, Mexico, Republic of Moldova, Nepal, and Peru). In 2 countries, the lag time was 4 or fewer weeks (France and the United States).

Using linear interpolation for missing data (Model 2; S3 Fig), the week of the first positive outlier changed in 7 countries. Among these countries, this week still predated the first reported COVID-19 peak (S2 Table). Compared to Model 1, the first outlier occurred earlier using linear interpolation in 3 countries (Bolivia, France, and Poland). In 4 countries, the outlier occurred later (Brazil, Peru, the Philippines, and Spain). With a linear interpolation approach, the earliest outliers were detected in Poland, on December 30, 2019 (seasonal level shift), and in Peru on January 20, 2020 (temporary change outlier). Using Model 2, on average outliers were detected 12.1 weeks prior to the first reported COVID-19 peak (standard deviation 7.8).

### Model fit

S4 and S5 Figs depict the autocorrelation function and partial autocorrelation function for residuals. Based on the Ljung–Box test, 6 countries demonstrated significant residual autocorrelation with a *p*-value less than 0.05, indicating potential issues with model fit: Afghanistan, Bangladesh, Colombia, Indonesia, Spain, and the United States (S3 Table).

## Discussion

This proof-of-concept study suggests the potential to leverage existing global influenza surveillance systems as early warning systems for national and global detection of the circulation of novel respiratory viral pathogens. Using a data-driven approach to identify outliers in influenza-negative ILI during the first year of the COVID-19 pandemic, we detected outliers in 16 countries that predated the first reported peaks of SARS-CoV-2. On average, detected outliers occurred more than 13 weeks before reported peaks. The use of automated outlier detection systems in global respiratory virus surveillance could have informed countries of the potential spread of SARS-CoV-2 and influenced early public health responses to mitigate subsequent disease spread and burden.

To our knowledge, this is the first study to evaluate trends in influenza-negative ILI in multiple countries globally during the COVID-19 pandemic. We identified several countries in which the cases of influenza-negative ILI in 2020 substantially exceeded historical trends. For instance, in Brazil, Mexico, Mozambique, and Peru, the observed numbers of cases were greater than 2-fold higher than observations over the preceding 5 years. The median number of influenza-negative ILI cases between 2015 and 2020 was highest in HICs, which may be indicative of the relative size of the surveillance systems. Missing observations of influenza-negative ILI were highly variable by country. In France, missing data were clustered in the summer months. In several other countries, including Brazil, India, and Mozambique, missing observations occurred primarily during 2020 after the onset of the pandemic, which may demonstrate the impact of the pandemic on routine surveillance systems.

The earliest outliers that were identified in our primary analysis occurred during the week of January 13, 2020, in Poland, Peru, the Philippines, and Spain. However, in our sensitivity analysis using linear interpolation for missing data, the first outlier shifted to March in 2 of these 4 countries. In Poland and Peru, the first laboratory-confirmed SARS-CoV-2 cases occurred in March 2020, whereas we detected outliers in influenza-negative ILI in these countries during the weeks of December 30, 2019, and January 20, 2020, respectively [19].

Comparing reported COVID-19 peaks to outliers in influenza-negative ILI, we observed that time differences were especially pronounced in upper- and lower-middle-income countries. For example, in Peru, the first outlier, which was detected in January 2020 using both our primary and interpolated model, occurred 7 weeks before the first reported COVID-19 case (March 2, 2020) and 20 weeks before the first COVID-19 peak (June 1, 2020). In many LMICs, including Bolivia, Colombia, and Mozambique, the first outlier occurred near the time of the first reported COVID-19 case and several weeks prior to the first peak. The observed lag times are likely impacted by both the robustness of public health reporting structures as well as local policies. For instance, limited availability of testing early in the pandemic may have resulted in underreporting of cases and delayed policy measures. Estimates of excess mortality for COVID-19 have suggested that the number of cumulative deaths was much higher than the number of reported deaths in many LMICs, including India, Russia, Mexico, Brazil, Indonesia, and Pakistan [25].

In 3 countries (Germany, Madagascar, and Uganda), the first detected outlier in 2020 occurred after the first reported COVID-19 peak. There are likely several contributing factors. For instance in Uganda, the relatively small size of the first COVID-19 wave and limited local transmission may have resulted in fewer cases in routine surveillance [26]. In Madagascar, there was limited community transmission through April, and the first outbreak occurred at a mining company [27]. SARS-CoV-2 positivity detected through ILI surveillance did not increase until June 2020 [27]. Notably, in all 3 countries the first detected outlier nonetheless appears to coincide with a subsequent outbreak of SARS-CoV-2 (Figs 5 and 8).

In previous single-country studies, influenza surveillance systems have been used to detect surges in ILI during the pandemic [9,10,28], and some studies have used laboratory testing to evaluate the incidence of SARS-CoV-2 among cases of ILI [12,28]. In the United States, a surge of non-influenza ILI was seen in March 2020, with a high positivity rate of SARS-CoV-2 among ILI cases beginning the week of March 8 [12]. These findings support the results of our study, which found an influenza-negative ILI outlier in the United States the week of March 9, 2020. In Italy and France, studies also demonstrated an excess of ILI cases in early 2020 [10,28]. Laboratory-based surveillance in Italy found a 71% positivity rate for SARS-CoV-2 among ILI cases during the week of March 9, 2020 [11]. Another study that evaluated changes in ILI trends and COVID-19 incidence and deaths in the United States, found that a peak in ILI *z*-scores preceded a rise in COVID-19 cases or deaths with a 2-week lag, which was attributed to the course of disease and testing delays [29]. Despite employing different methodologies, these studies support the concept that routine influenza surveillance data and data-driven monitoring approaches can be integral components of early disease detection systems in select settings.

While we cannot definitively conclude from our analysis that non-influenza ILI outliers were caused by undetected cases of SARS-CoV-2, our findings suggest that early community transmission of SARS-CoV-2 may have been underestimated in several countries. Alternatively, outliers may also reflect changes in case reporting or patterns in individuals seeking care for respiratory illness, particularly after a pandemic was declared. Disruptions to routine surveillance, due to the pandemic or other factors, may also impact these findings. For instance, in Mozambique, armed conflicts and displacements may have resulted in disruptions to disease surveillance [30]. The identified outliers may also represent circulation of other non-influenza respiratory viruses, particularly in the early months of 2020. After March 2020, the prevalence of many common respiratory viruses decreased, likely related to interventions to mitigate the spread of SARS-CoV-2 [31]. Lastly, the detected outliers may also represent false positives related to poor model fit.

There are several additional limitations to this study. First, a key assumption of ARIMA modeling is that the data are stationary, with a constant mean and variance. While differencing is conducted to achieve stationarity during the automated ARIMA process, some countries may continue to exhibit non-stationarity. In our model diagnostics, residuals from 6 countries demonstrated ongoing autocorrelation after model fit (S3 Table). If this method were to be used outside of this proof-of-concept analysis, the relative merits of fitting automated ARIMA models versus non-automated methods would have to be weighed. Second, in this study we are unable to quantify the false positive rate of detected outliers. For instance, in Poland, many detected outliers appear to coincide with seasonal peaks of ILI between 2015 and 2020. In other countries, such as France, the United States, Brazil, and Mexico, few positive outliers were detected in the series prior to the COVID-19 pandemic in 2020. In the United States, the outlier detected during 2018 coincided with a particularly high influenza burden [32]. To apply similar methodology in a real-world setting, it would be crucial to optimize model fit for each country and to assess the likelihood of a disease outbreak with each outlier. We envision that this system—if applied in a real-world setting—would represent an early warning sign that would indicate the need for further exploration and analysis to examine whether further public health action is needed to mitigate a potential outbreak of a known or unknown disease. In addition, the sensitivity of the model in detecting outliers can be modulated so that countries could determine their own optimal sensitivity for detecting potential outbreaks. Third, delays in surveillance system reporting may potentially limit the utility of this approach. Improving both data quality and reporting times for routine surveillance systems would be essential. Fourth, in this study the definition used for COVID-19 peaks may introduce a source of bias and impact the reported lag times. Country-level data reporting throughout the pandemic may have also been impacted by changing availability of testing and case definitions, resulting in under- or overestimation.

Despite these limitations, the use of data-driven approaches for outlier detection in routine surveillance systems may have broad applicability in the future. A major strength of this study is the use of automated ARIMA modeling, which allows for differences in temporal and seasonal patterns by country. Integrating automated outlier detection systems within surveillance networks and establishing multi-pathogen testing sites may be beneficial in detecting novel disease outbreaks. While data quality and data missingness are significant challenges, this study emphasizes the importance of strengthening reporting and data quality to maximize the public health impact of these routine surveillance systems. While we restricted the analysis in this study to countries with limited missing data, in our sensitivity analysis, the timing of identified outliers changed in 7 countries when we incorporated interpolated data into the models. Improving data quality and completeness for respiratory surveillance systems would likely enhance the feasibility of using data-driven approaches to detect deviations in trends over time.

In summary, this study demonstrates a potential role for automated outlier detection in the identification and monitoring of novel respiratory pathogens using influenza surveillance networks. During the first year of the COVID-19 pandemic, we found outliers in influenza-negative ILI for 16 countries that preceded reported COVID-19 peaks and, in some countries, the first reported COVID-19 case. In many LMICs the lag between the first outlier and the COVID-19 peak exceeded 12 weeks, which may be related to challenges in diagnostic testing, reporting, and public health responses during the early stages of the COVID-19 pandemic. Developing and improving existing respiratory disease surveillance networks will be essential for improving our response to future pandemics and limiting morbidity and mortality globally. An automated outlier detection system in respiratory disease surveillance could facilitate early identification of novel pathogens and inform public health responses including implementing multi-pathogen diagnostic testing, and enacting policies to mitigate the impact of disease outbreaks.

## Supporting information

S1 Fig Observed trends in influenza-negative ILI for countries without positive outliers in 2020. (DOCX)

S2 Fig Observed trends in COVID-19 for countries without positive outliers in 2020. (DOCX)

S3 Fig Trends using linear interpolation for missing data in time series by country. (DOCX)

S4 Fig Autocorrelation function for fitted time series model residuals. (DOCX)

S5 Fig Partial autocorrelation function for fitted time series model residuals. (DOCX)

S1 Table Missing data for influenza-negative ILI by country. (DOCX)

S2 Table Time series outliers by country using linear interpolation for missing data. (DOCX)

S3 Table Fitted time series model and Ljung–Box test for residuals. (DOCX)

S1 Text Countries with highest cumulative COVID-19 cases by January 3, 2021, stratified by World Bank income group. (DOCX)

S2 Text Calculation of overall completeness score based on missing data. (DOCX)

S3 Text Time series outliers by country for 2015–2020. (DOCX)

## Abbreviations

ARIMA: autoregressive integrated moving average
GISRS: Global Influenza Surveillance and Response System
HICs: high-income countries
ILI: influenza-like illness
ISO: International Organization for Standardization
LMICs: low- and middle-income countries
