# Supplementary material for: Global influenza surveillance systems to detect the spread of influenza-negative influenza-like illness during the COVID-19 pandemic: Time series outlier analyses from 2015–2020
Source: PLoS Med. 2022 Jul 19;19(7):e1004035. doi: 10.1371/journal.pmed.1004035 (PMC9295997; doi:10.1371/journal.pmed.1004035)
Supplement: S1 Fig — (DOCX) [file pmed.1004035.s001.docx]

**S1 Fig: Observed trends in influenza-negative ILI for countries without positive outliers in 2020**

**
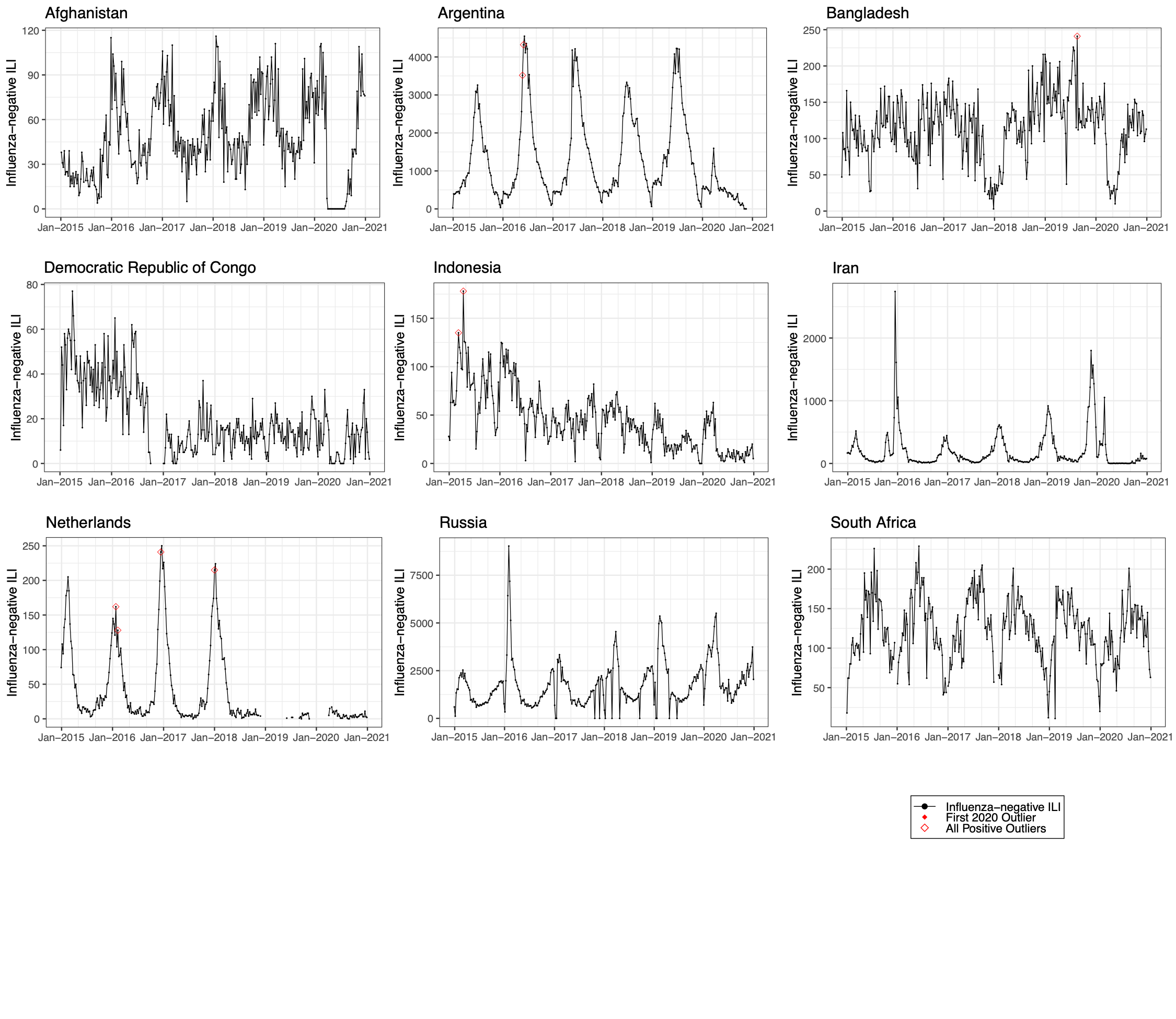
**

Legend: The figures display the reported cases of influenza-negative influenza-like-illness (ILI) and all detected positive outliers (red outline) in each country between 2015-2020.
