# Supplementary material for: Global influenza surveillance systems to detect the spread of influenza-negative influenza-like illness during the COVID-19 pandemic: Time series outlier analyses from 2015–2020
Source: PLoS Med. 2022 Jul 19;19(7):e1004035. doi: 10.1371/journal.pmed.1004035 (PMC9295997; doi:10.1371/journal.pmed.1004035)
Supplement: S2 Fig — (DOCX) [file pmed.1004035.s002.docx]

**S2 Fig: Observed trends in COVID-19 for countries without positive outliers in 2020**


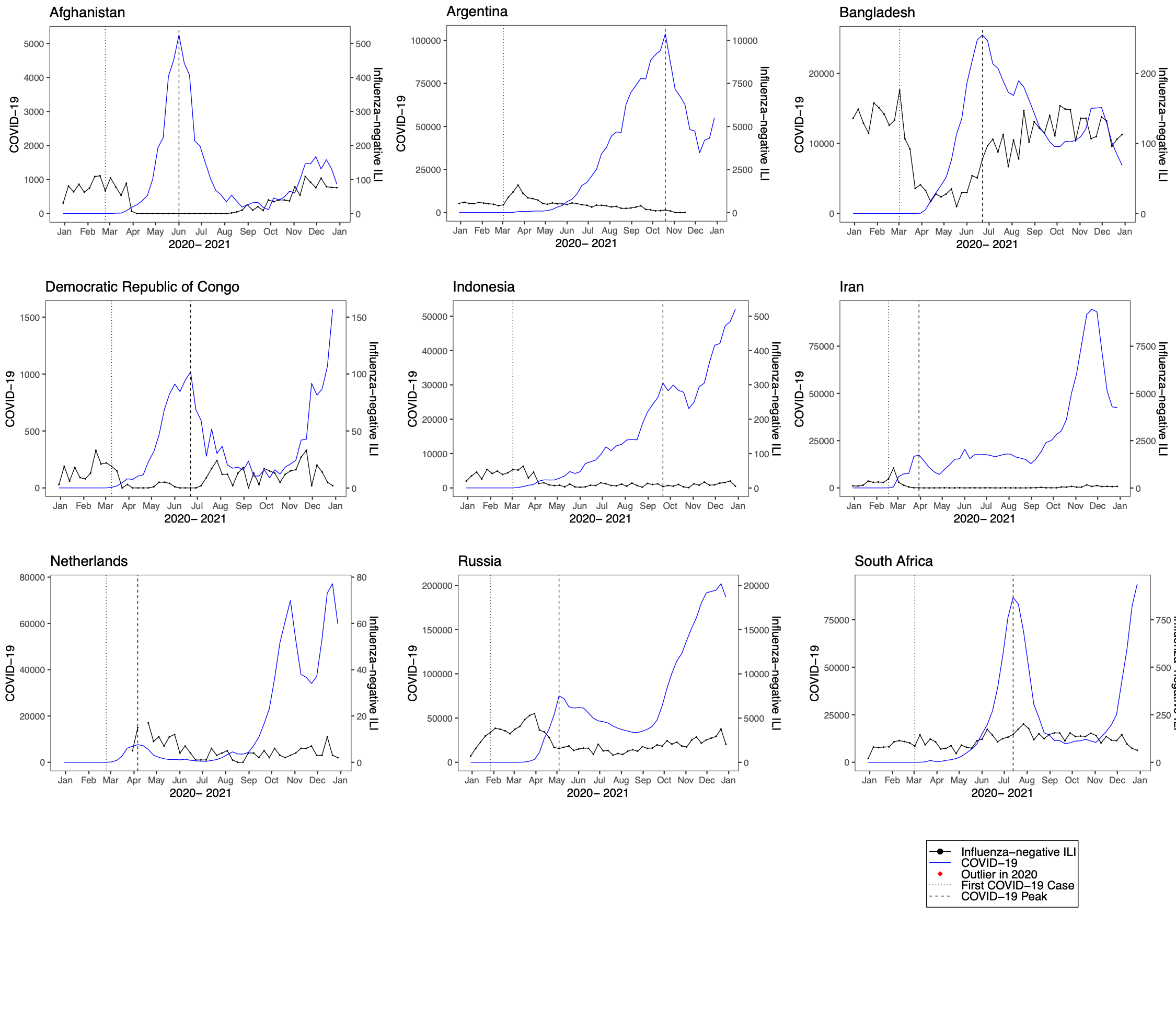


Legend. The figures show the trends in reported COVID-19 cases during 2020 (blue line), the week of the first COVID-19 case (vertical dotted black line) and the week of the first COVID-19 peak (vertical dashed black line).
