# Supplementary material for: Global influenza surveillance systems to detect the spread of influenza-negative influenza-like illness during the COVID-19 pandemic: Time series outlier analyses from 2015–2020
Source: PLoS Med. 2022 Jul 19;19(7):e1004035. doi: 10.1371/journal.pmed.1004035 (PMC9295997; doi:10.1371/journal.pmed.1004035)
Supplement: S3 Fig — (DOCX) [file pmed.1004035.s003.docx]

**S3 Fig:** **Trends using linear interpolation for missing data in time-series by country**

S3A: High- income countries


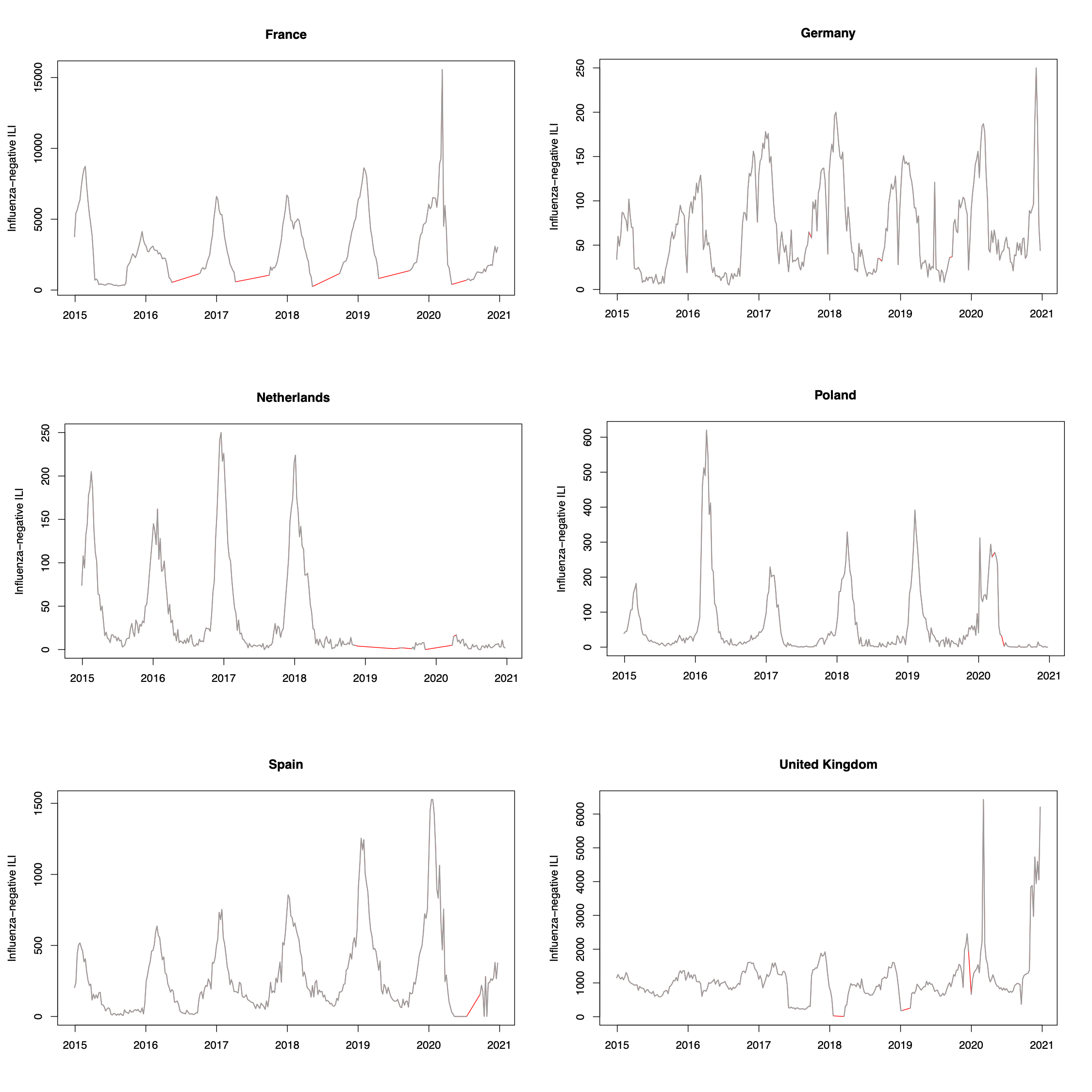


S3B: Upper-middle income countries


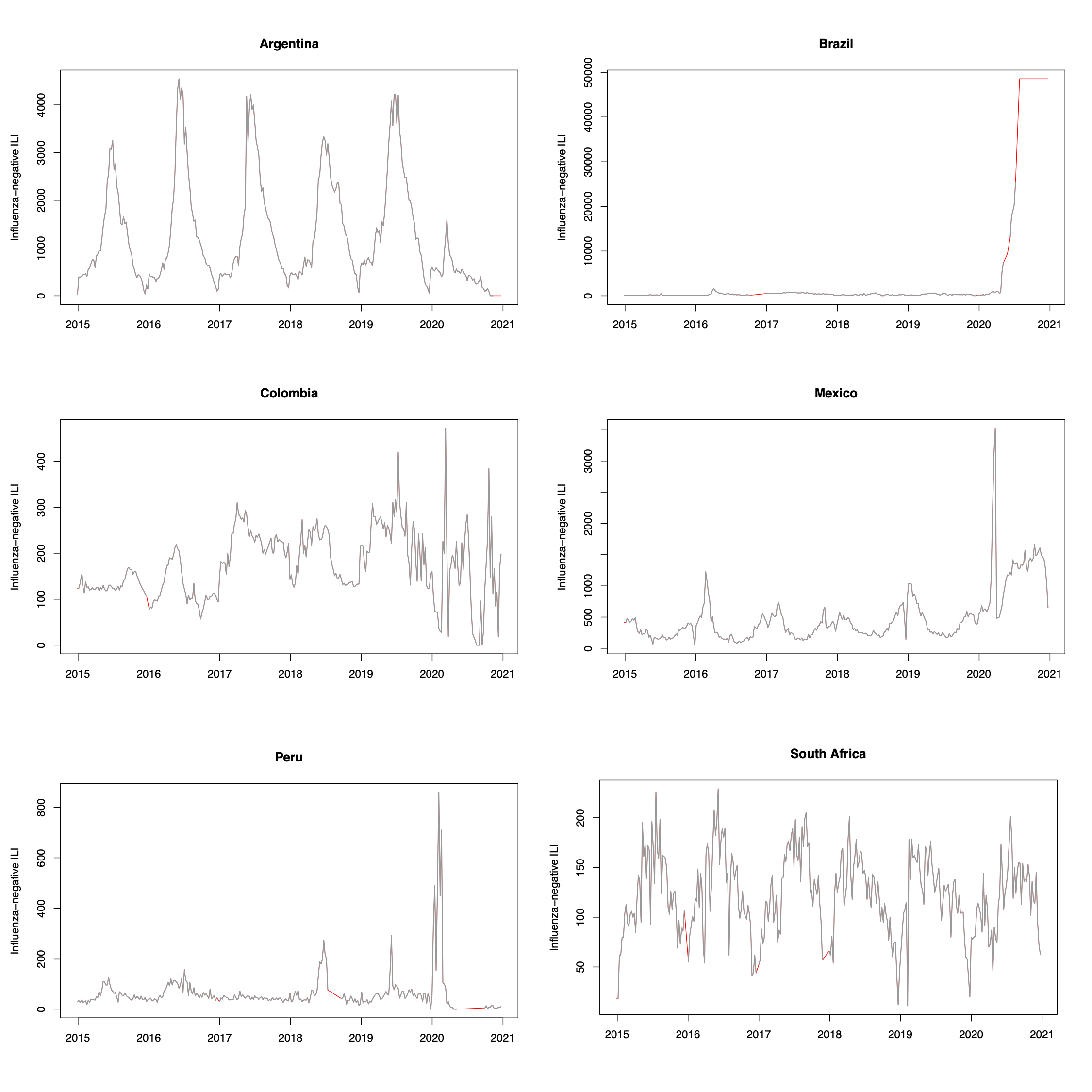


S3C: Lower-middle income countries

**
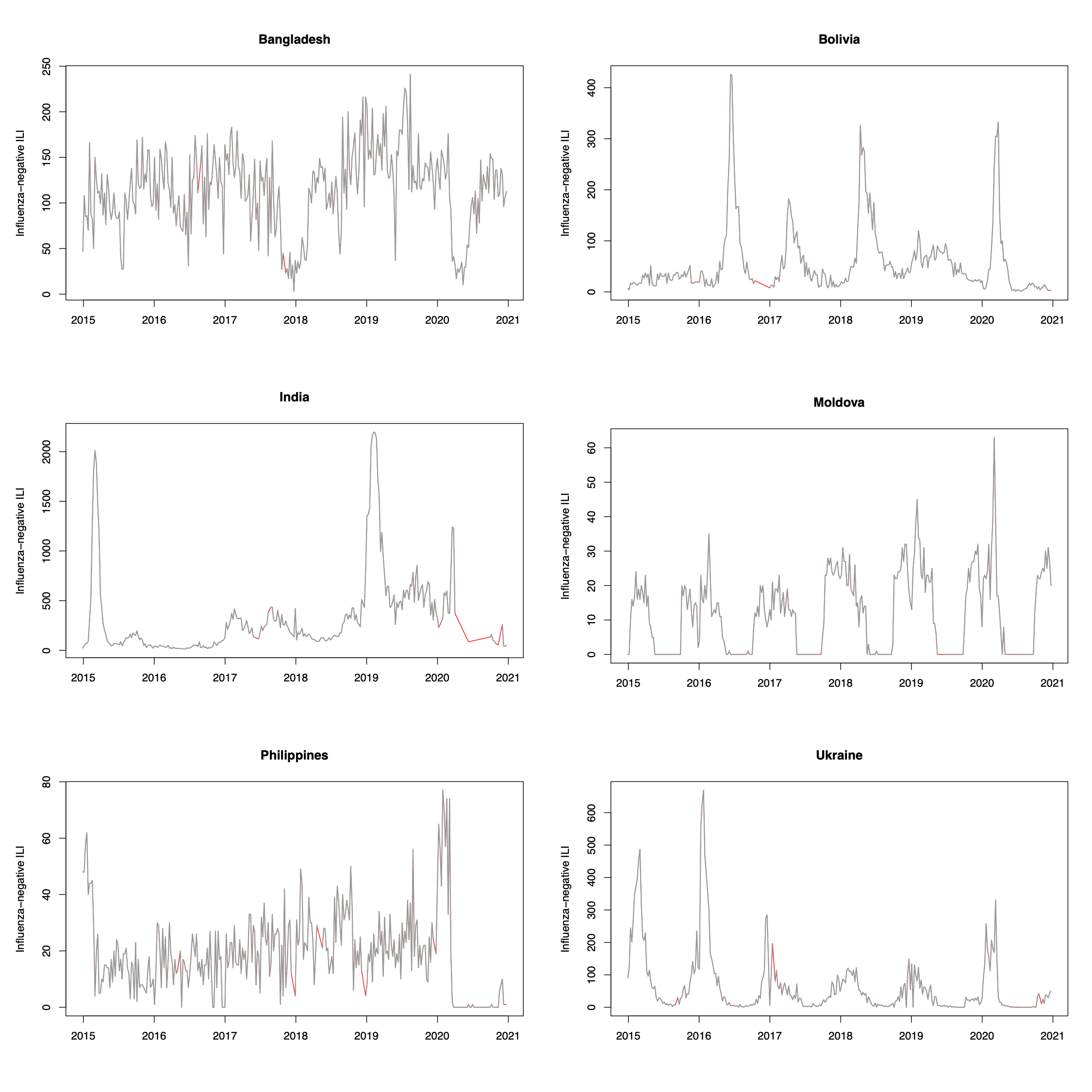
**

S3D: Low- income countries


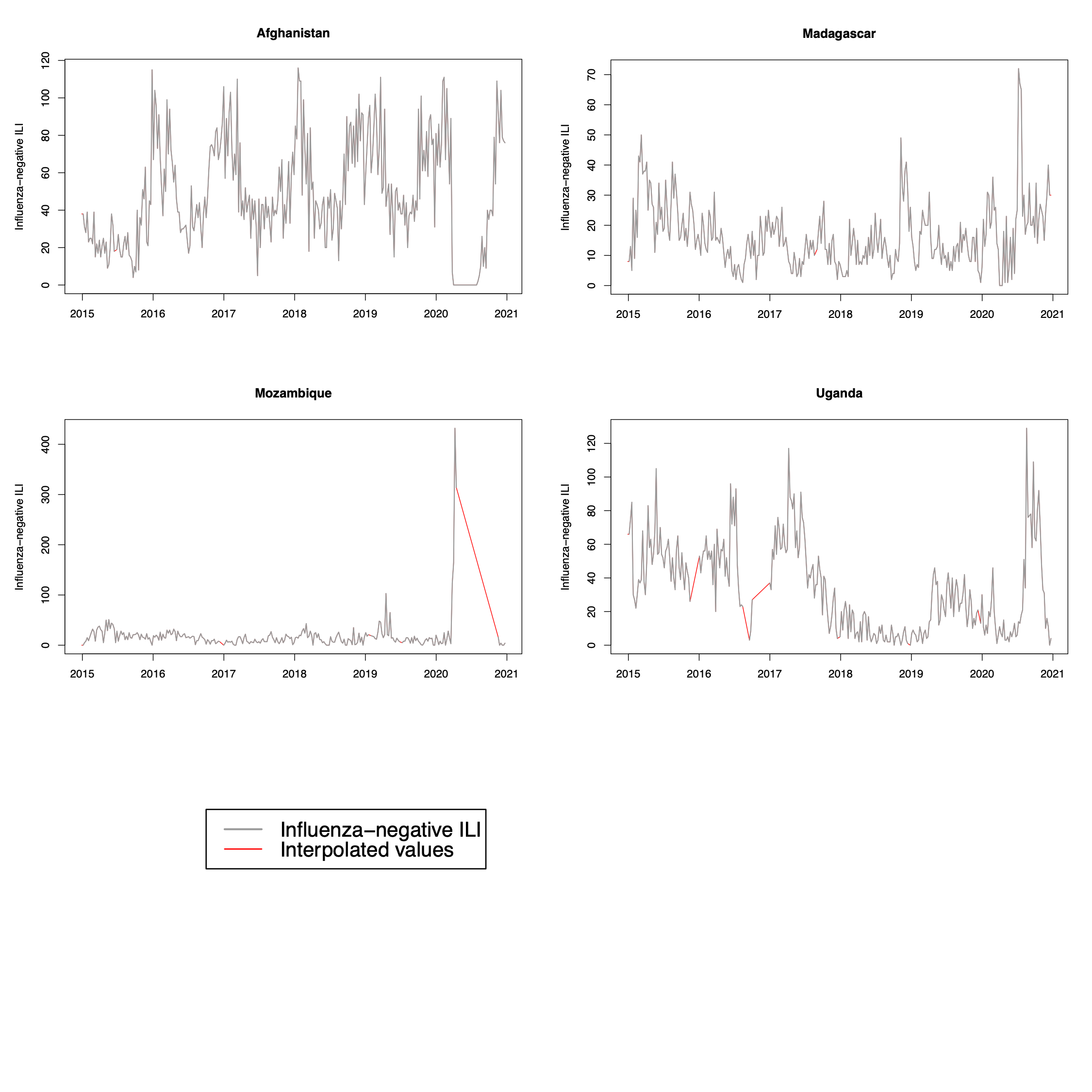


Legend: For each country, the graph shows the observed cases of influenza-negative influenza-like-illness (ILI) and interpolated values (bright red line) for missing data.
