# Supplementary material for: Global influenza surveillance systems to detect the spread of influenza-negative influenza-like illness during the COVID-19 pandemic: Time series outlier analyses from 2015–2020
Source: PLoS Med. 2022 Jul 19;19(7):e1004035. doi: 10.1371/journal.pmed.1004035 (PMC9295997; doi:10.1371/journal.pmed.1004035)
Supplement: S5 Fig — (DOCX) [file pmed.1004035.s005.docx]

**S5 Fig: Partial Autocorrelation Function for Fitted Time Series Model Residuals**

**
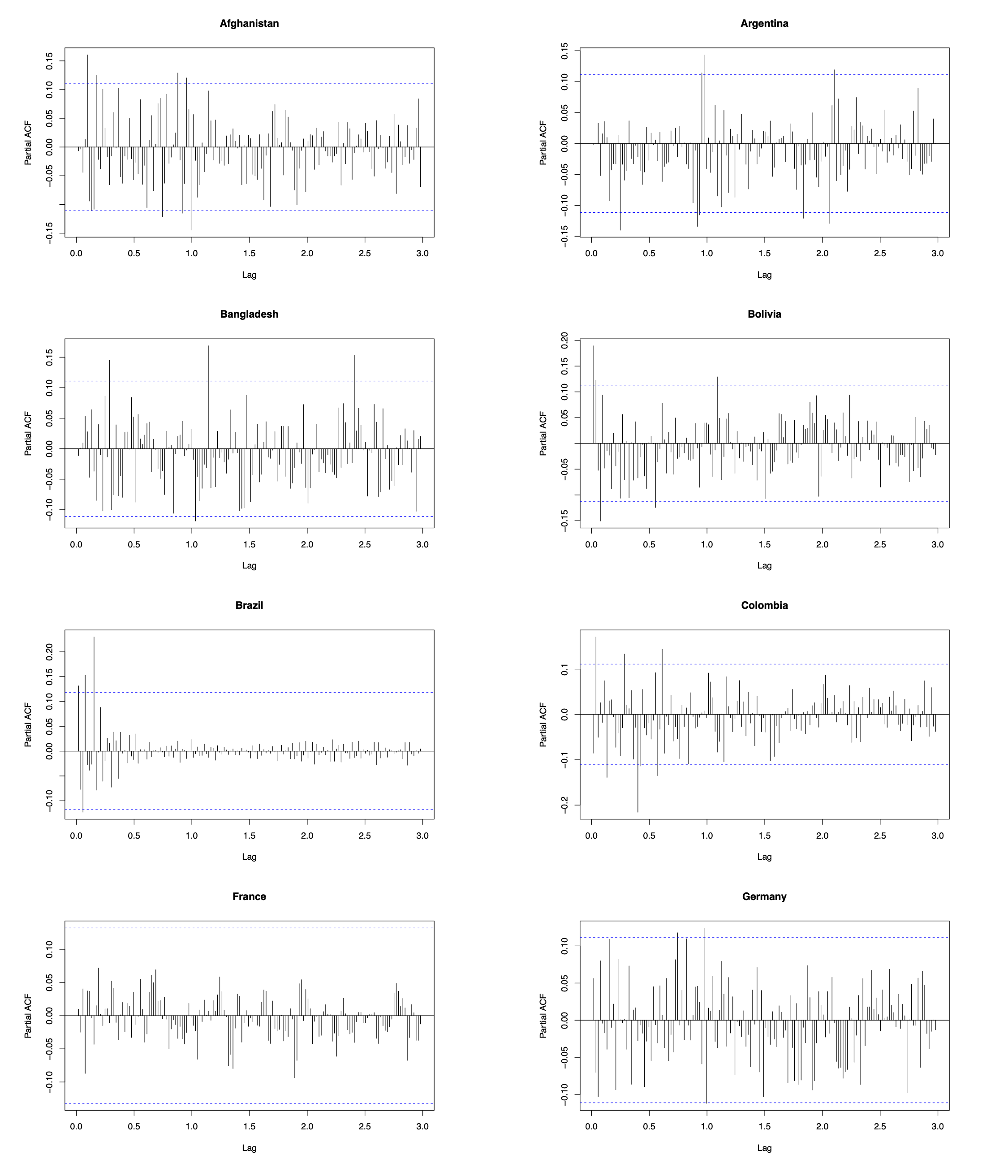
**


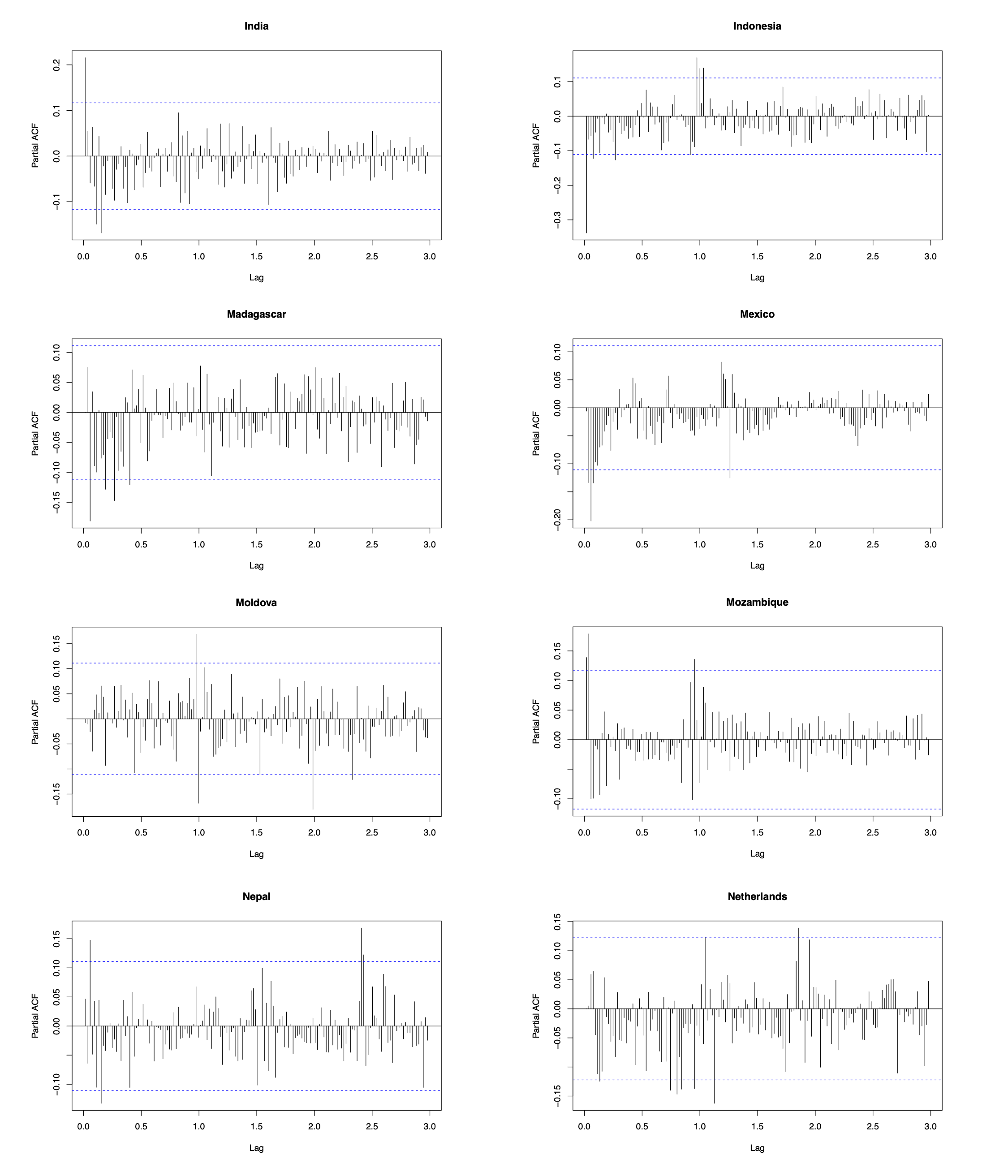


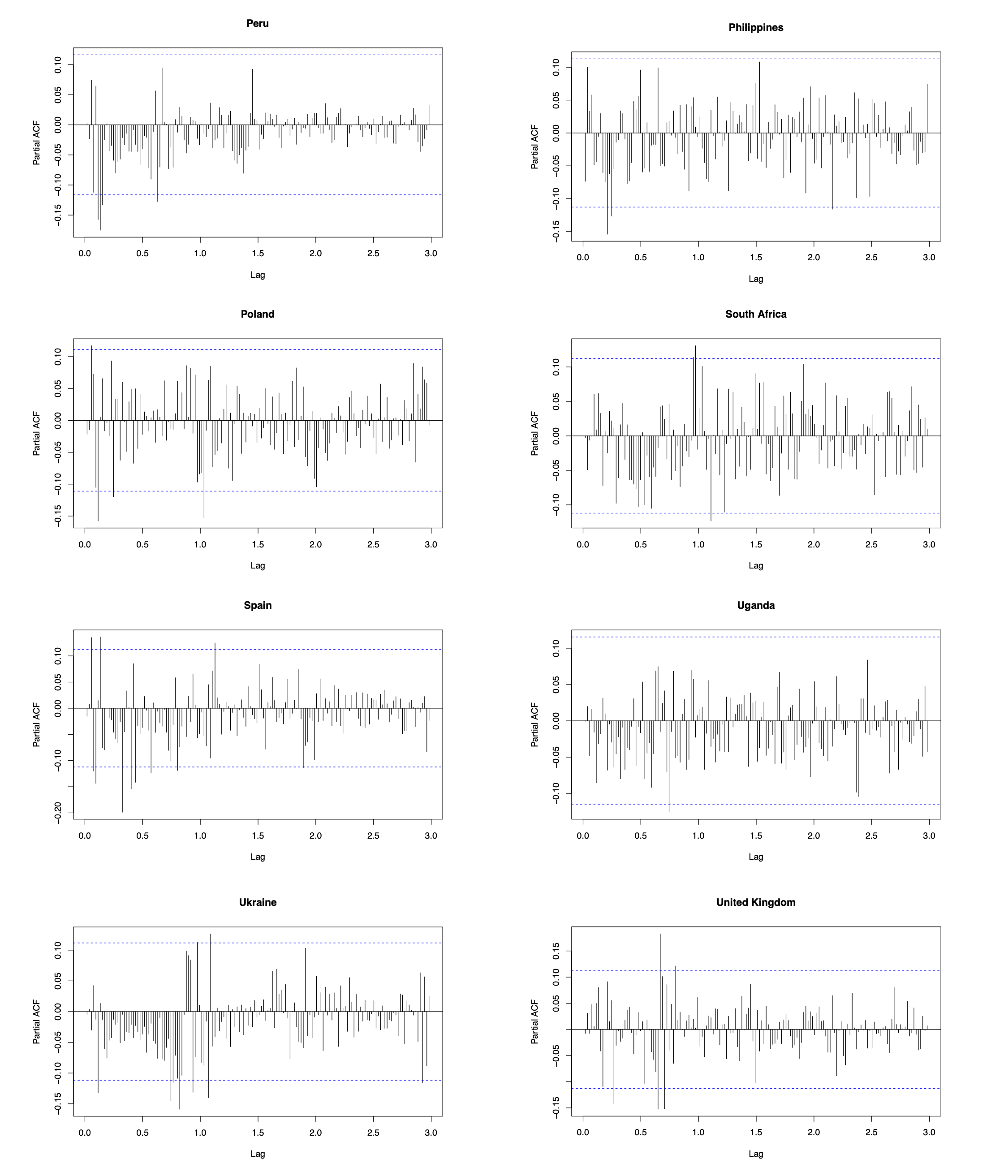


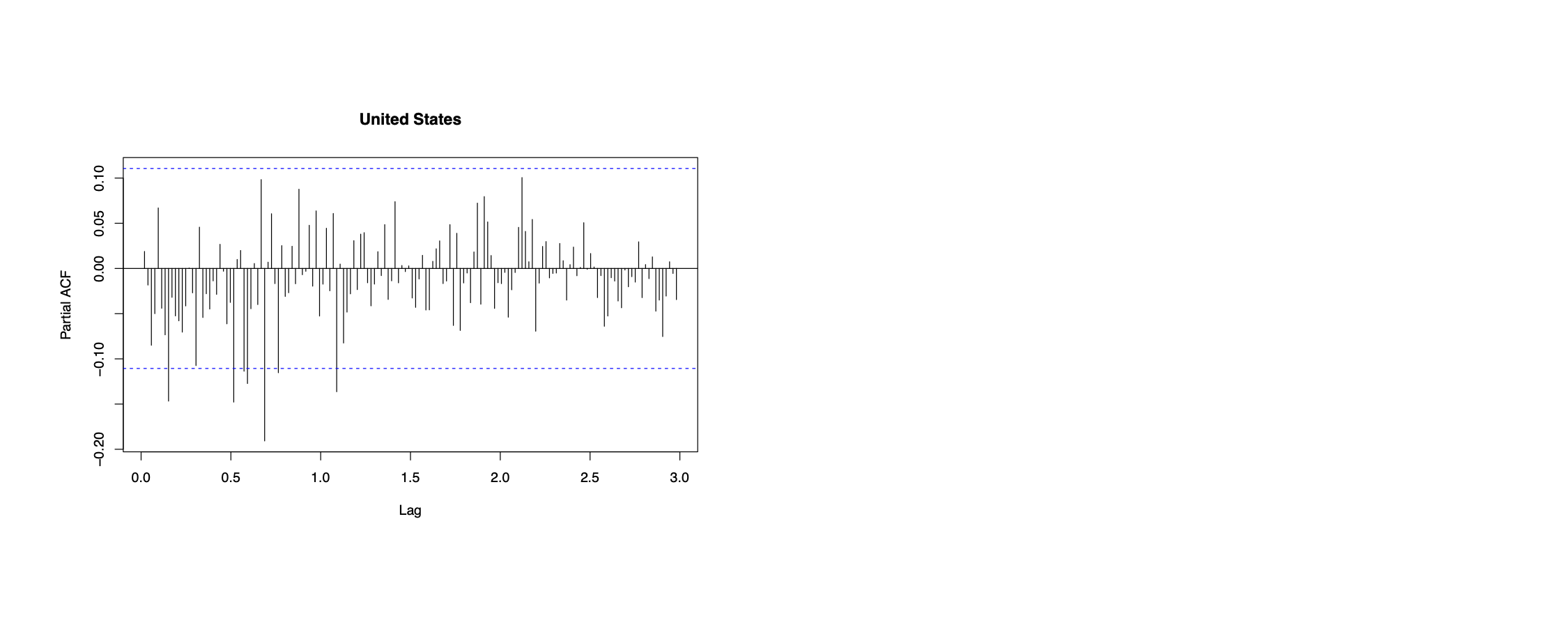


Legend: The figures show the partial autocorrelation function (pACF) of fitted Model 1 residuals for each country with the ninety-five percent confidence intervals (dashed blue line).
