# Supplementary material for: Global influenza surveillance systems to detect the spread of influenza-negative influenza-like illness during the COVID-19 pandemic: Time series outlier analyses from 2015–2020
Source: PLoS Med. 2022 Jul 19;19(7):e1004035. doi: 10.1371/journal.pmed.1004035 (PMC9295997; doi:10.1371/journal.pmed.1004035)
Supplement: S1 Table — (DOCX) [file pmed.1004035.s006.docx]

**S1 Table: Missing data for influenza-negative ILI by country**

| **Country** | **Influenza-negative ILI missing, count (%)** | **Longest gap, count^a^** | **Number of gaps, count^b^** |
| --- | --- | --- | --- |
| **HICs** |  |  |  |
| France | 94 (29.9) | 24 | 5 |
| Germany | 3 (1.0) | 1 | 3 |
| Netherlands | 57 (18.2) | 26 | 6 |
| Poland | 2 (0.6) | 1 | 2 |
| Spain | 9 (2.9) | 9 | 1 |
| United Kingdom | 13 (4.1) | 7 | 3 |
| United States | 0 | -- | -- |
| **U-MICs** |  |  |  |
| Argentina | 6 (1.9) | 6 | 1 |
| Brazil | 38 (12.1) | 21 | 6 |
| Columbia | 2 (0.6) | 1 | 2 |
| Indonesia | 0 | -- | -- |
| Iran | 0 | -- | -- |
| Mexico | 1 (0.3) | 1 | 1 |
| Peru | 30 (9.5) | 20 | 3 |
| Russia | 0 | -- | -- |
| South Africa | 8 (2.6) | 4 | 4 |
| **L-MICs** |  |  |  |
| Bangladesh | 2 (0.6) | 1 | 2 |
| Bolivia | 14 (4.5) | 10 | 3 |
| India | 32 (10.2) | 15 | 8 |
| Republic of Moldova | 4 (1.3) | 1 | 4 |
| Nepal | 0 | -- | -- |
| Philippines | 12 (3.8) | 3 | 7 |
| Ukraine | 6 (1.9) | 2 | 5 |
| **LICs** |  |  |  |
| Afghanistan | 2 (0.6) | 1 | 2 |
| DRC | 14 (4.5) | 12 | 3 |
| Madagascar | 3 (1.0) | 1 | 3 |
| Mozambique | 35 (11.1) | 30 | 5 |
| Uganda | 26 (8.28) | 12 | 7 |

**^a^** Longest gap refers to the highest number of consecutive missing observations for a given country over the entire time series from 2015- 2020.

**^b^** Number of gaps refers to the number of time periods that contain missing data for a given country over the entire time series trend from 2015- 2020.

HICs = High-income countries, U-MICs = Upper-middle income countries, L-MICs = Lower-middle income countries, LICs = Low-income countries

ILI = Influenza-like illness
