# Supplementary material for: Global influenza surveillance systems to detect the spread of influenza-negative influenza-like illness during the COVID-19 pandemic: Time series outlier analyses from 2015–2020
Source: PLoS Med. 2022 Jul 19;19(7):e1004035. doi: 10.1371/journal.pmed.1004035 (PMC9295997; doi:10.1371/journal.pmed.1004035)
Supplement: S2 Table — (DOCX) [file pmed.1004035.s007.docx]

**S2 Table**: **Time-series outliers by country using linear interpolation for missing data**

| **Country** | **Week of Detected Time Series Outlier (type)^a^** | **Week of First Reported COVID-19 Case** | **Week of First Reported COVID-19 Peak** | **Time Difference between Outlier and Peak (weeks)** |
| --- | --- | --- | --- | --- |
| **HICs** |  |  |  |  |
| France | 2-Mar-20  (AO) | 20-Jan-20 | 30-Mar-2020 | 4 |
| Poland | 30-Dec-2019 (SLS) | 2-Mar-20 | 6-Apr-2020 | 14 |
| Spain | 2-Mar-20  (AO) | 20-Jan-20 | 23-Mar-2020 | 3 |
| **U-MICs** |  |  |  |  |
| Brazil | 22-Jun-20  (TC) | 24-Feb-20 | 27-Jul-2020 | 5 |
| Peru | 20-Jan-20  (TC) | 2-Mar-20 | 1-Jun-2020 | 19 |
| **L-MICs** |  |  |  |  |
| Bolivia | 9-Mar-20  (AO) | 9-Mar-20 | 3-Aug-2020 | 21 |
| Philippines | 9-Mar-20  (AO) | 27-Jan-20 | 30-Mar-2020 | 3 |

**^a^** Additive outliers (AO), seasonal level shift (SLS), temporary change outliers (TC)

Note: For countries not listed above, the first detected outlier in 2020 did not change when compared to Model 1.

HICs = High-income countries, U-MICs = Upper-middle income countries, L-MICs = Lower-middle income countries, LICs = Low-income countries
