# Supplementary material for: Global influenza surveillance systems to detect the spread of influenza-negative influenza-like illness during the COVID-19 pandemic: Time series outlier analyses from 2015–2020
Source: PLoS Med. 2022 Jul 19;19(7):e1004035. doi: 10.1371/journal.pmed.1004035 (PMC9295997; doi:10.1371/journal.pmed.1004035)
Supplement: S3 Table — (DOCX) [file pmed.1004035.s008.docx]

**S3 Table: Fitted Time Series Model and Ljung-Box Test for Residuals**

| **Country** | **Fitted ARIMA Model (p, d, q)^a^, (P, D, Q)^b^** | **p-value** |
| --- | --- | --- |
| **HICs** |  |  |
| France | (2,0,4) | 0.999 |
| Germany | (1,1,1), (0,1,1) | 0.077 |
| Netherlands | (3,1,0), (1,0,0) | 0.738 |
| Poland | (3,0,0), (1,1,0) | 0.152 |
| Spain | (1,1,0), (1,1,0) | **<0.001** |
| United Kingdom | (1,0,1) | 0.102 |
| United States | (1,1,0), (0,1,1) | **0.031** |
| **U-MICs** |  |  |
| Argentina | (2,1,2), (1,1,1) | 0.722 |
| Brazil | (1,1,0) | 0.991 |
| Colombia | (1,1,1) | **<0.001** |
| Indonesia | (0,1,0), (2,0,0) | **<0.001** |
| Mexico | (0,1,1), (0,0,1) | 0.993 |
| Peru | (0,1,2) | 0.807 |
| South Africa | (1,0,1), (1,0,0) | 0.261 |
| **L-MICs** |  |  |
| Bangladesh | (4,0,2) | **0.015** |
| Bolivia | (1,0,0) | 0.318 |
| India | (0,1,0), (1,0,1) | 0.112 |
| Republic of Moldova | (4,0,0), (1,1,0) | 0.150 |
| Nepal | (1,0,0), (1,0,0) | 0.997 |
| Philippines | (0,1,1) | 0.336 |
| Ukraine | (3,1,1), (1,0,0) | 0.062 |
| **LICs** |  |  |
| Afghanistan | (2,1,2), (1,1,0) | **<0.001** |
| Madagascar | (0,1,1), (1,0,0) | 0.313 |
| Mozambique | (0,1,2), (1,0,0) | 0.699 |
| Uganda | (0,1,1), (1,0,0) | 0.898 |

^a^ Non-seasonal autoregressive term, difference term, and moving average term = (p, d, q)

^b^ Seasonal autoregressive term, difference term, and moving average term = (P, D, Q)

ARIMA= Autoregressive integrated moving average

HICs = High-income countries, U-MICs = Upper-middle income countries, L-MICs = Lower-middle income countries, LICs = Low-income countries
