# Supplementary material for: Global influenza surveillance systems to detect the spread of influenza-negative influenza-like illness during the COVID-19 pandemic: Time series outlier analyses from 2015–2020
Source: PLoS Med. 2022 Jul 19;19(7):e1004035. doi: 10.1371/journal.pmed.1004035 (PMC9295997; doi:10.1371/journal.pmed.1004035)
Supplement: S1 Text — (DOCX) [file pmed.1004035.s009.docx]

**S1 Text: Countries with highest cumulative COVID-19 cases by January 3, 2021, stratified by World Bank income group**

High-Income Countries (HIC**)**: Belgium, Czechia, France, Germany, Italy, Netherlands, Poland, Spain, United Kingdom of Great Britain and Northern Ireland, United States of America.

Upper-Middle Income Countries (U-MIC): Argentina, Brazil, Colombia, Indonesia, Iran, Mexico, Peru, Russian Federation, South Africa, Turkey

Lower-Middle Income Countries (L-MIC): Bangladesh, Bolivia, India, Morocco, Nepal, Pakistan, Philippines, Republic of Moldova, Ukraine, West Bank and Gaza Strip

Low-Income Countries (LIC): Afghanistan, Democratic Republic of Congo, Ethiopia, Guinea, Madagascar, Mozambique, Sudan, Syrian Arab Republic, Tajikistan, Uganda
