# Supplementary material for: Global influenza surveillance systems to detect the spread of influenza-negative influenza-like illness during the COVID-19 pandemic: Time series outlier analyses from 2015–2020
Source: PLoS Med. 2022 Jul 19;19(7):e1004035. doi: 10.1371/journal.pmed.1004035 (PMC9295997; doi:10.1371/journal.pmed.1004035)
Supplement: S2 Text — (DOCX) [file pmed.1004035.s010.docx]

**S2 Text:** **Calculation of overall completeness score based on missing data**

1. Percent complete for influenza positive = Number of observations for influenza positive specimens ÷ total weeks × 100
2. Percent complete for specimens processed = Number of observations for specimens processed ÷ total weeks × 100
3. Percent complete for specimens processed in 2020 = Number of observations for specimens processed in 2020 ÷ weeks in 2020 × 100

Percent complete overall = (a + b + c) ÷ 3
