# Supplementary material for: Global influenza surveillance systems to detect the spread of influenza-negative influenza-like illness during the COVID-19 pandemic: Time series outlier analyses from 2015–2020
Source: PLoS Med. 2022 Jul 19;19(7):e1004035. doi: 10.1371/journal.pmed.1004035 (PMC9295997; doi:10.1371/journal.pmed.1004035)
Supplement: S3 Text — (DOCX) [file pmed.1004035.s011.docx]

**S3 Text: Time Series Outliers by Country from 2015-2020**

Note: The first outlier detected in 2020 for each country is in bold.

High-Income Countries (HIC)

France: **9-Mar-2020**

Germany: 1-Jul-2019, **30-Nov-2020**, 7-Dec-2020

Netherlands: 25-Jan-2016, 8-Feb-2016, 12-Dec-2016, 1-Jan-2018

Poland: 8-Feb-2016, 29-Feb-2016, 21-Mar-2016, 23-Jan-2017, 26-Feb-2018, 11-Feb-2019, **13-Jan-2020**

Spain: 21-Jan-2019, 4-Feb-2019, **13-Jan-2020**, 27-Jan-2020, 2-Mar-2020, 23-Mar-2020

United Kingdom: 2-Dec-2019, **9-Mar-2020**, 9-Nov-2020, 30-Nov-2020, 14-Dec-2020, 28-Dec-2020

United States: 22-Jan-2018, **9-Mar-2020**, 16-Mar-2020, 2-Nov-2020, 9-Nov-2020, 30-Nov-2020

Upper-Middle Income Countries (U-MIC)

Argentina: 23-May-2016, 30-May-2016

Brazil: **4-May-2020,** 22-Jun-2020**,** 13-Jul-2020**,** 3-Aug-2020

Colombia: 15-Jul-2019, 26-Aug-2019, **2-Mar-2020**, 16-Mar-2020, 14-Sep-2020, 26-Oct-2020

Indonesia: 9-Mar-2015, 13-Apr-2015

Mexico: 13-Mar-2017, **23-Mar-2020**, 30-Mar-2020

Peru: 25-Jun-2018, 10-Jun-2019, **13-Jan-2020**, 20-Jan-2020, 10-Feb-2020, 24-Feb-2020

South Africa: None

Lower-Middle Income Countries (L-MIC)

Bangladesh: 19-Aug-2019

Bolivia: 30-May-2016, 6-Jun-2016, 13-Jun-2016, 20-Jun-2016, 16-Apr-2018, 23-Apr-2018, 11-Mar-2019, **16-Mar-2020**, 30-Mar-2020

India: 23-Feb-2015, 7-Jan-2019, 28-Jan-2019, **23-Mar-2020**

Moldova: 8-Oct-2018, 4-Feb-2019, 11-Mar-2019, **9-Mar-2020**

Nepal: 2-Mar-2015, 29-Aug-2016, 31-Jul-2017, 21-Jan-2019, 18-Feb-2019, **10-Feb-2020**, 16-Mar-2020

Philippines: 5-Jan-2015, 12-Jan-2015, 2-Sep-2019, **13-Jan-2020**, 3-Feb-2020

Ukraine: 12-Jan-2015, 2-Mar-2015, 11-Jan-2016, 12-Dec-2016, **16-Mar-2020**

Low-Income Countries (LIC)

Afghanistan: None

Madagascar: 23-Feb-2015, 12-Nov-2018, **13-Jul-2020**

Mozambique: 5-Nov-2018, 22-Apr-2019, 13-May-2019, **30-Mar-2020**, 6-Apr-2020, 13-Apr-2020

Uganda: 25-May-2015, 13-Jun-2016, 11-Jul-2016, 10-Apr-2017, 12-Jun-2017, **24-Aug-2020**, 28-Sep-2020, 19-Oct-2020
